# Racial Disparities in Breast Cancer Treatments and Adverse Events in the SEER-Medicare Data

**DOI:** 10.3390/cancers15174333

**Published:** 2023-08-30

**Authors:** Robert Wieder, Nabil Adam

**Affiliations:** 1 Rutgers New Jersey Medical School and the Cancer Institute of New Jersey, 185 South Orange Avenue, MSB F671, Newark, NJ 07103, USA; 2 Phalcon, LLC, Manhasset, NY 11030, USA; n.adam@phalconllc.com

**Keywords:** breast cancer treatment, chemotherapy, hormone therapy, biological therapy, racial disparities, adverse events, SEER-Medicare

## Abstract

**Simple Summary:**

Breast cancer (BC) occurs more frequently in white (W) women than in African American (AA) women, but AA women die at a greater rate from BC than W women. Disparities exist in all aspects of the disease, from screening and early detection, to access to and completion of treatment. There are reports that adverse events from treatment also occur more frequently in AA patients. Here, we showed differences in treatments administered to AA women using the SEER-Medicare dataset. We found that AA women receive universally less treatment in every category of cancer and stage in the setting of the National Claims History database of Oncology practice providers. The drugs used to treat BC were relatively similar between the two races. Still, there were small differences in rates of administration of drugs that caused higher rates of side effects, which caused higher overall rates of adverse events in AA patients. The data suggest that the setting where AA patients receive care affects their treatment rate and adverse events. It is possible that higher rates of recorded adverse events result in lower rates of treatment or completion of treatment in AA women with BC.

**Abstract:**

Despite lower incidence rates, African American (AA) patients have shorter survival from breast cancer (BC) than white (W) patients. Multiple factors contribute to decreased survival, including screening disparities, later presentation, and access to care. Disparities in adverse events (AEs) may contribute to delayed or incomplete treatment, earlier recurrence, and shortened survival. Here, we analyzed the SEER-Medicare dataset, which captures claims from a variety of venues, in order to determine whether the cancer care venues affect treatment and associated adverse events. We investigated a study population whose claims are included in the Outpatient files, consisting of hospital and healthcare facility venues, and a study population from the National Claims History (NCH) files, consisting of claims from physicians, office practices, and other non-institutional providers. We demonstrated statistically and substantively significant venue-specific differences in treatment rates, drugs administered, and AEs from treatments between AA and W patients. We showed that AA patients in the NCH dataset received lower rates of treatment, but patients in the Outpatient dataset received higher rates of treatment than W patients. The rates of recorded AEs per treatment were higher in the NCH setting than in the Outpatient setting in all patients. AEs were consistently higher in AA patients than in W patients. AA patients had higher comorbidity indices and were younger than W patients, but these variables did not appear to play roles in the AE differences. The frequency of specific anticancer drugs administered in cancer- and venue-specific circumstances and their associated AEs varied between AA and W patients. The higher AE rates were due to slightly higher frequencies in the administration of drugs with higher associated AE rates in AA patients than in W patients. Our investigations demonstrate significant differences in treatment rates and associated AEs between AA and W patients with BC, depending on the venues of care, likely contributing to differences in outcomes.

## 1. Introduction

The median survival with breast cancer (BC) is shorter in African American (AA) patients than in white (W) patients, despite a lower incidence of the disease in AAs [1,2,3,4]. There are well-documented social factors that affect survival, including disparities in screening, diagnosis, and treatment [5,6]. Biologic differences in the molecular characteristics of the cancers also contribute to disparities between the two populations [7,8]. Molecular differences in cancer cells include variations in gene isoforms and expression patterns, cancer stem cell content and characteristics, microRNAs, tRNA-derived fragments, hypoxia-inducible factors, and epigenetics [9,10,11,12,13,14,15,16,17,18]. Differences in cellular, genetic, epigenetic, inflammatory, and immune factors and vitamin D content in the tumor microenvironment also determine differences in BC’s aggressiveness and treatment resistance in AA patients [18,19,20]. Recognition of these molecular differences has directed investigations towards therapeutic approaches to race-specific molecular targets, the exceptional intra- and inter-tumor heterogeneity of triple-negative cancer, and the non-genetic risk factors that can alter genetic and epigenetic programs [21,22,23,24,25].

Treatment disparities that contribute to survival differences include delays in seeking care [5], delays in initiating care [26,27,28], administration of lower dose proportions or dose intensity of prescribed therapy [29,30,31], race-dependent differences in different venues [26,28], lower rates of receiving treatment and of appropriate treatment according to the National Comprehensive Cancer Network practice guidelines, and many others [5,6,32]. Significant societal efforts to ameliorate social disparities have resulted in a glimmer of hope of shrinking the differences in rates of screening [33]. The differences in survival of patients with hormone-negative localized disease have also narrowed, likely from closing the gap in the administration of standard-of-care treatment in this highly aggressive disease [34]. Nevertheless, disparities in survival and every other measure of treatment success remain unabated.

One factor contributing to the undertreatment of AA BC patients is early termination of therapy due to adverse events (AEs) caused by treatment. Treatment-induced AEs impose significant obstacles to tolerating therapy, quality of life, and the ability to administer adequate therapy, a factor that affects outcomes [35]. A large retrospective review of 12 published articles from four large databases concluded that AAs were significantly more likely to delay the initiation of therapy, to have dose-density reductions, and to discontinue treatment than W patients with early-stage BC [36]. This has been attributed to differences in the trigger points applied by treating oncologists to reduce or change drugs or stop treatment, or to differences in the frequency of AEs in different circumstances [36]. However, despite more frequent rates of incomplete or delayed treatment of AA patients due to AEs, only a few drugs, including anthracyclines [37] and taxanes [37,38], cause specific differences in AEs experienced by AA compared with W BC patients. In fact, several studies have reported no statistically significant differences in AEs between AA and W patients induced by chemotherapy drugs [1,29,39]. At the same time, some population-averaged investigations only showed a greater incidence of mild AEs and only in later treatment cycles in AA patients [40].

Comprehensive data have not been generated on the differential rates of use of available drugs for systemic therapy of different molecular classifications of BC and different stages of disease between the two races and different venues of care. Hence, the rates of AE-generating treatments in different scenarios could not be evaluated. Understanding these treatment-specific rates could aid decision-making in specific circumstances of medical practice to decrease the high likelihood of AEs in specific groups of patients. Most available studies are small, are generated from retrospective cohorts, have not been investigated systematically, and often report no differences among the races in population-averaged data [37,41]. Here, we systematically investigate the Surveillance, Epidemiology and End Results (SEER)-Medicare-linked (S-M) dataset over the period 1991–2016 to identify the differences in cancer and patient variables associated with different applications of BC therapy in two divergently different care-providing venues, the Outpatient dataset from hospital and healthcare facility venues and the NCH files from non-institutional providers. Our goal was to identify rates of treatment and specific classes of drugs inducing differences in AEs between patients from the two races. We intended to determine whether the venue of care is responsible for treatment and AE differences between the two populations.

## 2. Materials and Methods

### 2.1. Generation of Database

We used the S-M dataset, which we obtained through a two-tiered review process under an IRB exempt-review protocol [42]. Medicare files included in the S-M dataset capture the fee-for-service claims from hospitals (MEDPAR), outpatient facilities, NCH, hospice care, home health agencies (HHA), the Outpatient dataset (consisting of hospital and healthcare facility venues), and Part D Prescription Drug Event (PDE) claims, starting in 2007. It is important to note here that NCH records are physician/supplier (Part B) bills for 100% of all claims that are collected by Centers for Medicare and Medicaid Services (CMS) and are from physicians and office practices, although it includes claims from other non-institutional providers such as physician assistants, clinical social workers, nurse practitioners, independent clinical laboratories, ambulance providers, and stand-alone ambulatory surgical centers.

For this study, we analyzed patient records from the NCH and Outpatient datasets over the period 1991–2016. Each Medicare claim file includes the unique patient identifier and typically includes the date(s) of service, diagnosis codes, procedure codes, and the amount charged/reimbursed. The Medicare CCflag file includes, for every patient, the date the patient was diagnosed with one of 22 chronic conditions; each of these chronic conditions has a certain weight [43]. Data that included medical diagnoses were derived from 1991 to 2015 and included only ICD9 codes, as ICD10 codes only came into use on 1 October 2015.

### 2.2. Generation of a Patient Cohort That Met Our Selection Criteria

We included women who had a valid patient identification number (ID), were diagnosed with BC with no other malignancy by NCI clinical trials definitions [44], stage (I, II, III, or IV), valid sex, date of diagnosis, and date of birth. In general, in the case of a missing day of the month, we assumed the first day of the month. We removed duplicate records in the NCH and Outpatient datasets to generate our working datasets. We divided patients into two categories for analysis: stages I–III and stage IV. We considered patients as hormone receptor-positive if they were recorded as estrogen receptor (ER)+ or progesterone receptor (PR)+ and hormone receptor-negative if they were recorded as ER− and PR−. Patients’ Human Epidermal Growth Factor Receptor (Her)2 status was considered if they were categorically recorded as Her2− or Her2+. We included all comorbidities, all prior treatments, delineated age, race, marital status, BC stage, grade, hormone receptor and Her2 status, and laterality. A visit was included if it had a valid ID and date, diagnosis, procedure, or Healthcare Common Procedure Coding System (HCPCS) code. We generated a dataset from the NCH files that included 289,727 BC patients, with 37,672,583 entries, 32,699,642 visits, and 706,971 administered treatments. We also generated a dataset from the Outpatient files that included 252,478 BC patients, with 8,034,700 entries, 7,044,636 visits, and 269,025 administered treatments.

We identified 141 HCPCS drug J codes: 82 chemotherapy drugs, 49 biotherapy drugs, and ten hormone therapy drugs. We included licensed drugs used or investigated in BC clinical trials from a Medline search. We consolidated the drugs into 46 categories and one no-treatment category by mechanisms of action, such as alkylating agents, antimetabolites, anthracyclines, taxanes, bisphosphonates, growth factors, monoclonal antibodies and small molecules, hormone receptor inhibitors, for example, and generated AE data. We counted individual treatments multiple times, each time a 0, 1, 2, ..., n AE was recorded.

To generate a metric of the wellness of the patients from the available data, we tracked the same diagnostic codes and consolidated categories we used to determine treatment-associated AEs and catalogued them to determine their frequency in patients when not temporally associated with treatment. We considered these non-treatment-associated AE codes abnormal medical conditions and considered them an indicator of patient health.

We consolidated AEs into related categories to enable an easier presentation and correlation with drug categories, cancer molecular markers, and stage variables associated with their occurrence. We included all International Classification of Diseases (ICD) codes (only ICD-9) of potential AEs in a category, regardless of rarity, to ensure the capture of all events. These broad categories, representing cancer treatment AEs in the literature, are (0) no-adverse events, (1) infection or fever, (2) neutropenia or leukopenia, (3) thrombocytopenias, (4) anemias, (5) electrolyte abnormalities, (6) liver or gall bladder abnormalities, (7) weakness, malaise, or delirium, (8) nausea or vomiting, (9) diarrhea, (10) thrombophilia, (11) pulmonary embolus, (12) edema of any organs, (13) skin rashes, (14) weight loss or malnutrition, (15) respiratory symptoms, (16) constipation, (17) mucositis, and (18) neuropathy. We excluded chronic infections, HIV, positive TB tests, contacts, carriers, procedural or traumatic infections, neonatal or infantile infections, childhood, pregnancy, or parturition categories. We defined acute AEs most commonly associated with chemotherapy [20,35,45,46,47,48] by algorithms that flagged two events. One set included events that occurred from the day of treatment, which in practice can be induced by the administration of treatment (set 1 = (AE categories 1, 7, 8, 11–13, 15)), and a set that started from the day after treatment (set 2 = (AE categories 2–6, 9, 10, 14, 16–18)), both sets occurring within 21 days from the day of treatment and also if the given AEs did not occur within the 21 days before. We opted for 21 days because treatment-induced thrombocytopenia may take that long to recover [49]. Many AEs last much longer, and this approach excluded them. This time frame increased the likelihood that the AEs associated with a treatment were not already present at the time of the treatment [50].

### 2.3. Statistical Analysis

The significance of differences between any two groups was determined by a two-tailed *t*-test and assumed normal distributions, given the sample sizes [51]. We used Chi-square tests of association for data value distributions in categories. We rejected the null hypothesis for type I error with *p* values ≤ 0.05.

## 3. Results

### 3.1. Distributions of Patient Characteristics

Our initial observations of BC treatment in AA and W patients revealed differences in patient distributions, treatments, and AEs between the S-M Outpatient and NCH datasets. These two venues have distinct characteristics in location, organization, continuity of care, and distribution of care providers. These and other potential differences likely contribute to differences in treatments and outcomes in the patient populations they serve. Investigating combined data from the two venues may mask racial disparities in treatment and the incidence of AEs. Here, we examined the two datasets separately to discover relevant features associated with potential disparities.

First, we analyzed differences in patient distributions by race, age, and comorbidity index within the two datasets. The combined W and AA patients represented similar percentages in the two datasets. The Outpatient dataset contained 243,814 stages I–III patients, of which 94.3% were W and AA. The NCH dataset contained 279,814 stages I–III patients, of which 93.8% were W and AA. The remaining 6% belonged to other racial categories. There were 8664 stage IV patient entries in the Outpatient setting, of which 95.2% were W and AAs, and 9913 stage IV NCH patients, of which 94.9% were W and AA patients. The remaining 5% were patients recorded as being of other races.

We compared the patient distributions by stage and race in the Outpatient and NCH datasets (Table 1). Most patients were in the stage I group, and their frequency declined with progressive stages. AA patients were significantly less frequent in stage I and more frequent in progressive stages than W patients, with nearly identical percent distributions in the two S-M venue datasets. The percentage of AA patients in the pooled populations rose form 8.4% in stage I progressively to 15.7% in stage IV, nearly identically in both datasets. These results support the hypothesis that treatment and AE differences in the different datasets were not based on differences in race distributions.

Patient ages did not vary between the Outpatient and NCH datasets. W patients ranged in age between 67.5 and 69.5 years in the four cancer stages, while AA patients ranged from 62.2 to 66.0 in the four stages. AA patients were younger than W patients in every stage category (Table 2). Comorbidity indices were higher in the Outpatient dataset in both races at every stage. In addition, AA patients had higher comorbidity indices than W patients in every stage in both venue datasets (Table 2). Of note, comorbidity indices of stage IV patients in both the W and AA categories were lower than those of stage I, II, and III patients, respectively. We also analyzed a grouped dataset of stages I–III, comprising patients with localized disease, to characterize their ages and comorbidity indices as a ground reference for the grouped analysis that we undertook to determine treatment and AE differences. The age and comorbidity differences in the combined groups were similar to the individual stage I, II, and III groups.

### 3.2. Treatment and Adverse Events Distributions

The data revealed significant differences in treatment rates and treatment-associated AEs based on venue, race, and stage. All patients received more treatment with higher cancer stages. Comparing venues, all W patients of every stage received significantly more treatment in the NCH setting than in the Outpatient setting. AA patients received significantly more treatment in the Outpatient setting than in the NCH setting in stages I–III. Stage IV differences between the two venues were not statistically significant in AA patients.

**Figure 1:**
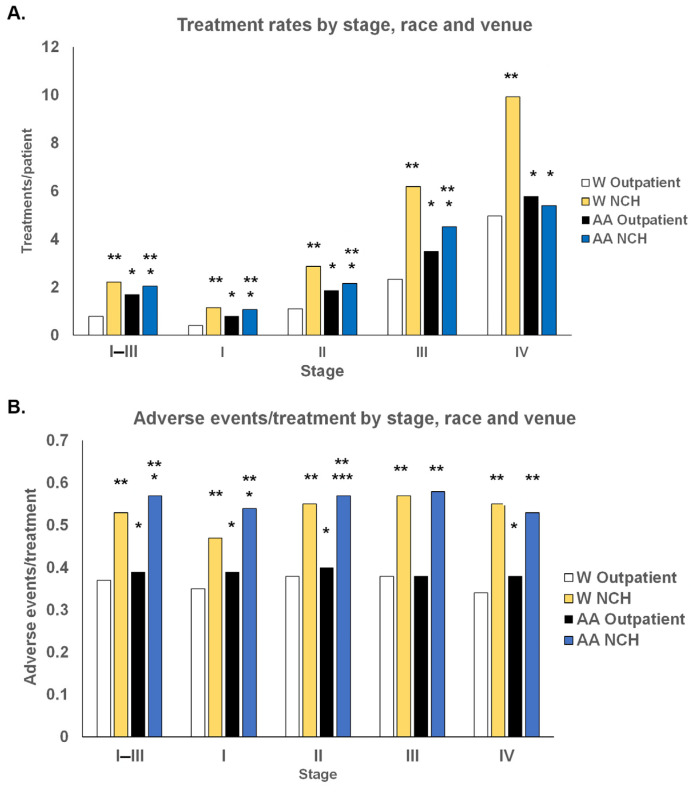
Differences between treatments/patient and adverse events/treatment by stage, race, and venue. (**A**) Statistically significant differences between treatment rates are shown between AA and W patients (* *p* = 0, Chi square) and between values in the NCH and Outpatient datasets (** *p* = 0, Chi square). (**B**) Statistically significant differences are shown between adverse events/treatment between AA and W patients (* *p* = 0, Chi square, *** *p* = 0.02, Chi square) and between NCH and Outpatient values (** *p* = 0, Chi square).

Race had a contrasting impact on treatment in the two venues (Figure 1A). AA patients received more treatment/patient than W patients in the Outpatient setting, while W patients received more treatment/patient than AA patients in the NCH setting. The preferential treatment ratio of AA/W patients in the Outpatient setting was 1.9-fold with stage I patients but gradually decreased to 1.17-fold in stage IV patients. This value was 2.13-fold higher in the combined stage I–III dataset. Analogously, the small 0.93-fold lower difference in the AA/W patient treatment rate in stage I patients in the NCH setting increased to a 0.54-fold lower AA/W treatment/patient ratio in stage IV patients.

Differences in the rates of AEs from treatment were observed based on venue, race, and in some instances, on stage, and were correlated in all significant instances with higher comorbidity indices in AA patients (Figure 1B). The rates of AEs per treatment were uniformly higher in the NCH dataset for both races and all stages, respectively. The rates of AEs/treatment were relatively uniform in both settings for both races across the four stages. The rates of AEs/treatment in the Outpatient setting were higher in AA patients than in W patients in stages I, II, and IV and in the NCH setting for stages I and II. In order to determine if potential reasons for these differences are associated with tumor types, we investigated the distinct BC populations defined by hormone receptor and Her2 cancer traits.

### 3.3. Effects of ER/PR and Her2 Status on Racial Differences in Treatments and AEs

We compared three-dimensional differences in the data between venues, racial categories, and cancer types (characterized by hormone and Her2 receptors for localized and stage IV disease). Our goal was to determine if higher rates of AEs were associated with these variables. The distribution of patients with ER/PR+ and ER/PR- tumors were identical in the Outpatient and in the NCH datasets, both in stages I–III and stage IV BC (Table 3 and Tables S1–S4). Analogously, patients with Her2− and Her2+ tumors were distributed in identical patterns in the two venues in both stages I–III and stage IV groups. All stage I–III ER- patients were younger than ER/PR+ patients, and all Her2+ patients were younger than Her2− patients. Stage IV ER/PR patient age differences were not statistically different in either venue. Some minimal age differences between venues were statistically but not clinically significant.

There were distinct differences between W and AA patients within both datasets. AA patients were universally younger in every category than W patients; AA patients had more frequent rates of ER/PR− and Her2+ patients than ER/PR+ and Her2− patients in both stage groups in both venues. Stage IV patient datasets had relatively few patients, and consequently, some differences, such as race-dependent age differences in ER/PR+ in the NCH setting, did not reach statistical significance.

Comorbidity indices were universally higher in the Outpatient dataset for all patients in the stage I–III category, stage IV W patients, ER/PR+, and Her2− AA patients with sufficient entries to generate statistically significant values. Comorbidity indices were also higher in stage I–III ER/PR+ than in ER/PR− patients in both W and AA patients in both venues. AA patients had significantly higher comorbidity indices than W patients in all stage I–III groups except in the Her2+ group, which had few entries. The stage IV differences were variable. These data add some granularity to the data on comorbidities in the unparsed population noted above. Specifically, ER/PR+ patients had higher comorbidity indices than ER/PR− patients, and AA patients had higher comorbidity indices in stage I–III cancers. The higher comorbidities in both races and venues in stage I–III ER/PR+ and Her2− cancers compared with ER/PR− and Her2+ cancers correlated with higher age. The pattern did not hold up in stage IV disease. In contrast, the higher comorbidity in stage IV AA patients than in W patients had an inverse relationship with age between the races, acknowledging that other known factors besides age account for this disparity in comorbidities between the races.

We categorized the differences in the treatment rates noted above by cancer type. Treatments per patient were significantly higher in patients with ER/PR− and Her2+ cancers than those with ER/PR+ and Her2− cancers, respectively, in both venues, stage categories, and races. In stage IV AA patients, these differences did not reach statistical significance due to small numbers. Patients in the NCH dataset received more treatment per patient in respective categories than in the Outpatient setting, except for one stage IV category with few patients. Racial differences in treatment were also evident. As noted in the pooled cancer dataset, AA patients received more treatment per patient than W patients in the Outpatient setting for both ER/PR+ and ER/PR− cancers and Her2− and Her2+ cancers and the converse; AA patients received less treatment per patient than W patients in every receptor category in the NCH setting. Similar reciprocal patterns were observed in the stage IV setting.

When analyzing the data by cancer receptor categories, AEs per treatment remained universally higher in the NCH setting than in the Outpatient setting. The differences in rates of AEs/treatment were more variable when we compared cancer types. In stage I–III BC, patients with ER/PR− cancers had higher rates of AEs than patients with ER/PR+ cancer in the Outpatient setting in both races. W patients in the NCH setting had a similar pattern. However, Her2− cancers inexplicably had higher rates of AE/treatment in both races in the stage I–III patients than the more aggressive Her2+ cancers. AE rates in stage IV disease generally did not follow a pattern. Racial differences in AEs/treatment were more complex. They were not evident in the stage I–III Outpatient setting and were only higher than those of W patients in NCH ER/PR+, Her2-, and Her+ tumors but not in ER/PR- tumors. In stage IV patients, the AA group had higher AEs in ER/PR+ and Her2- Outpatients, but no racial differences were noted in the NCH group.

These data confirm a portrait of different treatment rates and AEs per treatment between venues and patients of different races in the two venues. We proceeded to analyze specific treatments and AEs in the different stages and venues to identify drugs administered and specific AEs that may be associated with these observed rate differences.

### 3.4. Treatment Regimens and AEs

We compared the differences in the distribution of treatment and AE categories by stage, race, and venue. We compared stage I–III patients and stage IV patients in the two venues. We catalogued the ages and comorbidity indices of the specific group of patients who received the indicated treatments. We catalogued treatments and AEs recorded in at least 1% of cases.

The most frequent drug categories used in stages I–III and stage IV were similar in both venues, respectively, but administration rates varied by a few percentage points, moving them up or down by a few places on the list (Table 4 and Table 5). However, the most significant difference between treatments in the two venues was a much higher rate of AEs associated with the same drugs in the NCH setting than in the Outpatient setting in both stage categories, respectively. A group of drug categories that included anthracyclines, platinum compounds, and in most cases, pyrimidine analogs was associated with a consistent, conspicuous pattern of significantly higher rates of AEs than other drugs. Another group of drug categories that included bisphosphonates, folate analogs, antiestrogens, and VEGF inhibitors was associated with significantly lower rates of AEs in most categories. However, the order and frequency of their use did not cause the differences in the rates of AEs between the NCH and Outpatient settings.

We delineated the frequency rates of specific AE categories in the two venues in both stage I–III and stage IV patients. The same limited set of AEs made up the vast majority of AEs in both venues in both stage categories. However, their order and incidence rates varied greatly depending on the venue. In the adjuvant treatment setting in stage I–III patients (Table 6), the most distinct difference was a more than two-fold higher rate of nausea/vomiting in the NCH setting than in the Outpatient setting, with a frequency rate of 53%. Neutropenia/leukopenia occurred at nearly double the rate in the Outpatient setting than in the NCH setting. Weakness/malaise/delirium was 2.5 times more frequent in the Outpatient group than in the NCH category. In contrast, electrolyte abnormalities were recorded at double the rate in the NCH group than in the Outpatient dataset.

In stage IV patients, the pattern of frequency rate differences between venues was analogous to that of stage I–III patients. Nausea/vomiting was also two-fold more frequent in the NCH setting than in the Outpatient setting (Table 6). Electrolyte abnormalities were three times as frequent in the NCH setting than in the Outpatient setting. Weakness/malaise/delirium occurred at twice the rate in the Outpatient setting than in the NCH setting, and neutropenia occurred at nearly twice the rate in the Outpatient setting than in the NCH setting.

To try to understand the potential contributions of varying patterns of drug frequency use associated with these stark differences in the rates of specific AEs between the two venues, we determined the patterns of use of the most frequent drug categories associated with these differences in AE patterns in stages I–III and stage IV (Tables S5 and S6). We delineated the drugs most frequently used as treatments generating nausea/vomiting, weakness/malaise/delirium, neutropenia/leukopenia, and electrolyte abnormalities in stage I–III and IV patients. There were no stark differences in the distribution of use of these drug categories that could account for the numerical differences in the rates of AEs between the venue categories. Adding up the products of the percentages of AEs/treatment of particular treatments in the averaged populations from Table 4 and Table 5 and the treatment frequency in Tables S5 and S6 did not provide a clear rationale for the higher rates of AEs in the NCH population in either stage setting. Neither did the comorbidities of patients who received these treatments correlate with the AE rates. However, what became evident was that patients who experienced these AEs were uniformly older in the NCH category than patients in the Outpatient category in the stage I–III and stage IV groups (Tables S5 and S6). We proceeded to investigate the role of race in the distribution of AEs in the two venue categories.

### 3.5. Racial Effects of Treatments and AEs

Some features were evident when comparing W and AA patient treatments and AEs. In stage I–III patients in the Outpatient setting, the total percentage of AEs/treatment rates was minimally higher in AA patients than in W patients (Table 7A). Frequently used drug categories such as taxanes, Her2 targeting agents, alkylating agents, anthracyclines, pyrimidine analogs, and platinum compounds had small differences between the percentage of treatments generating AEs between the two racial categories and hence generated only small differences in the rates of AEs/TR, as their frequencies of use were slightly higher than those in W patients. The comorbidity indices of AA patients were uniformly higher than those of W patents, but their ages were uniformly lower. These data explain some of the observed lack of significant differences in AEs between the races in the stage I–III Outpatient dataset observed in Table 3.

**Figure 2:**
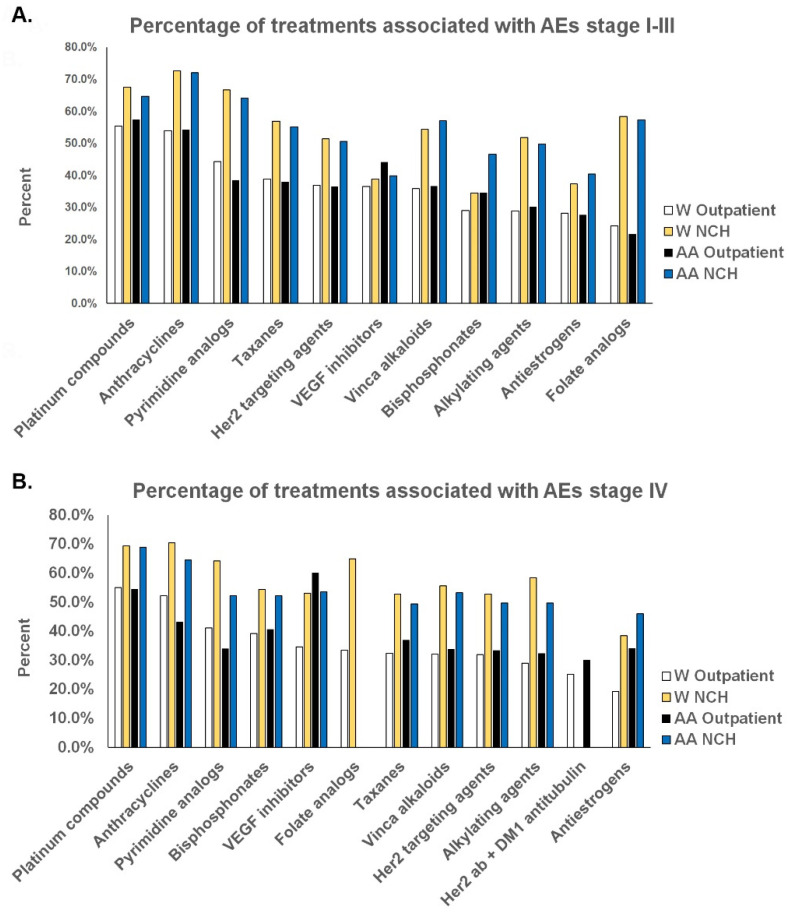
Differences between the rates of AEs associated with specific treatments in the Outpatient and NCH datasets. (**A**) Stage I–III patients and (**B**) stage IV patients treated with drugs comprising more than 98% of treatments had higher rates of AEs associated with most drug categories in the NCH setting than in the Outpatient setting in both racial categories.

However, as noted earlier in the whole population study, the percentages of drug treatments generating AEs were higher in the NCH setting than in the Outpatient setting for patients of both races (Figure 2). The differences in overall rates of AEs between the NCH and Outpatient settings resulted from the differences in the sum of the individual rates of AEs contributed by specific treatments in the two venues (highlighted with yellow backgrounds) (Table 7A,B). These individual rates represented the products of the frequency of use of a drug from the percent of the all drugs column and the percent of treatments with that drug resulting in AEs from Figure 2. Some of the most commonly used drugs, which had higher rates of inducing AEs than other drugs, were used with greater frequency in AA patients than in W patients. These combined factors accounted for the greater overall rates of AEs in stage IV AA patients than in W patients in the NCH setting. As in the Outpatient setting, the comorbidity indices of AA patients were universally higher than those of W patients, and their ages were universally lower in the NCH setting as well. It is, therefore, unlikely that these two characteristics can be attributed to being responsible for higher rates of AEs per treatment in the AA population in this setting.

The overall percentage of AEs per treatment in the stage IV Outpatient setting was somewhat higher in AA patients than in W patients (Table 7B). This mostly corresponded to higher rates of taxane used and associated AEs in AAs than W patients, as well as other treatments with higher rates of AEs, but at a more variable rate than in stage III patients. Of note, the comorbidity indices of AA patients in this category were not greater than those of W patients, but the AA patients were uniformly younger.

In the stage IV NCH setting, W patients had a higher rate of AEs per treatment than AA patients (Table 7B). This resulted mostly from several drugs that induced lower rates of AEs per treatment in AA patients than in W patients (Figure 2B). The comorbidities of AA patients were lower in this dataset than those of W patients.

The race and venue-based distributions of AEs confirmed our prior observations of an approximately two-fold higher rate of nausea and vomiting in the NCH venue than in the Outpatient venue (Tables S7 and S8). This was true for both races. Rates of weakness/malaise/delirium were higher in the Outpatient setting than in the NCH setting and consistently higher in both settings in W patients than AA patients. Electrolyte abnormalities were higher in the NCH setting for both races than in the Outpatient setting and were higher in AAs than W patients in the stage IV NCH setting only. Rates of neutropenias and anemias were higher in the Outpatient setting and always slightly higher in AA patients than in W patients. This may be associated with ethnic neutropenia not accounted for by caregivers [52]. Respiratory symptoms were higher in the Outpatient setting but relatively even between the races. Overall, these differences suggest that different AEs may be documented at different rates in different venues.

We reanalyzed the data using corrected definitions of Institutional Outpatient and Private Practive Office care venues using relevant CLM_TYPE, OPSRVTYP, plcsrvc, and fac_type codes, and derived analogous conclusions. The methods, results and conclusions are outined in Appendix A.

## 4. Discussion

The key message these investigations present is that determining disparities in treatment and AEs in AA patients must be done in carefully defined and controlled datasets because the venue of care has significant impacts on the results. Our investigations of the S-M datasets demonstrated that data from the Outpatient files generated different conclusions about the general treatment of breast cancer and AEs in the unparsed population and the AA and W populations than data from the NCH files. Published data on disparities in breast cancer treatment and AEs in minority populations are often based on studies from single centers or a relatively small number of patients that do not have sufficient power to generate stratified inferences on disparities in treatments and AEs that could be applied to whole populations [29,30,31,32]. Our investigations characterized data on the general population in the two S-M settings to establish a basis for comparing the W and AA populations in the context of their characteristics in the two venues.

Prima facie, the demographics of the two datasets appeared indistinguishable. Patient distributions by stage and race were identical and correlated with well-known characteristic differences between the two races [2,3,14,19,34]. AA patients were universally younger than W patients in every category and had lower rates of early-stage cancers and higher rates of more advanced-stage cancers than W patients. The distributions of patients with ER/PR+ and ER/PR− tumors and Her2− and Her2+ tumors were identical in the Outpatient and NCH datasets and in the stage I–III and stage IV BC categories. All stage I–III ER/PR− patients were younger than ER/PR+ patients, and all Her2+ patients were younger than Her2− patients. AA patients had more frequent rates of ER/PR− and Her2+ cancers than ER/PR+ and Her2− cancers, respectively, in both stage groups and venues. Some stage IV subgroups represented small percentages of patients and had few numbers, making the statistical evaluation of their significance difficult. By these criteria, it may appear reasonable to presume that data from the two datasets could be pooled for analysis to derive treatment and AE differences between the races.

However, on closer analysis, additional patient variables—such as comorbidity indices, treatments per patient, and AEs associated with treatments—yielded significant differences between the two venues. Comorbidities were higher in the Outpatient group for both races at every stage and with cancer hormone receptor and Her2 status binaries than in the NCH group. Comorbidity indices were also higher in stage I–III ER/PR+ than in ER/PR− patients in both W and AA patients in both venues. These higher comorbidities correlated with higher age in the NCH treatment settings, but the correlation was not sustained in other groups. For example, AA patients had significantly higher comorbidity indices than W patients in stage I–III groups, where there were sufficient entries to determine significance. Still, the ages of those patients were significantly lower than those of W patients in that group. Similarly, higher comorbidity rates of stage IV AA patients than ones in W patients had an inverse relationship with age. This was a key contradiction that underscored the well-documented differences in comorbidities between AA and W patients based on other medical and social factors [53,54,55,56] and downplayed the rationale for the role of comorbidities in explaining AE differences.

All patients received more treatment with higher cancer stages. Treatments per patient were significantly higher in patients with ER/PR− and Her2+ cancers than ER/PR+ and Her2− cancers, respectively, in both venues, both stage categories, and both races. Patients in the NCH dataset received more treatment per patient in correlative categories than patients in the Outpatient setting. W patients of every stage and receptor status received significantly more treatment in the NCH setting than AA patients. In comparison, AA patients received significantly more treatment in the Outpatient setting than W patients. These results underscore a fundamental difference in cancer care between the two racial groups in the two venues. They raise the prospect that differences in care and perception of AEs in the two venues have an outsized impact on the ultimate quantity and type of treatment, documentation of AEs, and the effect that perceived AEs weigh on continuing and completing treatment. Indeed, this is congruent with differences in cancer care between the two races in different venues that have been reported [55,57,58,59].

To make it clear, these data do not define the significance or meaning of receiving more care. Furthermore, we pooled drugs into categories to permit analysis of sufficient patient numbers per group, and we did not analyze doses and regimens in this study. Our investigation did not distinguish between the effects of less treatment on the completion of regimens in the adjuvant setting, nor on administration of multi-drug regimens. Additionally, the study did not approach the question of whether administration of additional treatments of successive regimens in stage IV breast cancer patients actually prolongs survival past the point of survival without the new treatment. The results presented here will permit these important follow-up investigations.

The rates of AEs per treatment were uniformly higher in the NCH dataset for both races and all stages, respectively. Patients with ER/PR− stage I–III cancers had higher rates of AEs than patients with ER/PR+ cancer in the Outpatient setting in both races. W patients in the NCH setting had a similar pattern. AA patients had higher rates of AEs/TR in the Outpatient and NCH setting for both ER/PR+ and ER/PR− tumors than W patients. The stage IV setting was less clear when broken down into tumor marker subcategories due to fewer entry numbers and more heterogeneous clinical scenarios.

The differences in AEs became clearer when the rates of specific drug-induced AEs were summed up for populations in each stage group for each race with sufficient entries to permit statistical analysis. These data showed that AA patients had small but consistently higher rates of AEs with treatment of stage I–III BC in both venues and stage IV BC in the Outpatient setting. Again, the opposite effect was documented in stage IV NCH patients, but its significance will have to be verified in larger datasets with more values in that category. We combined AEs into categories to permit sufficient values for statistical analysis, as we did with treatments. Hence, drug-specific AE relationships were not derived in this investigation.

The most frequent drug categories used in stages I–III and stage IV were similar in both venues. However, the most significant difference between treatments in the two venues was a much higher rate of AEs associated with the same drugs in the NCH setting than in the Outpatient setting in both stage categories, respectively, causing the observed differences in the overall rates of AEs in the two venues. The rates of induced AEs by individual drugs were similar between W and AA patients. However, some of the most commonly used drugs, associated with a consistent pattern of significantly higher rates of induced AEs than other drugs, moved up or down by a few places on the frequency of use list in most categories. A more frequent use of drugs with higher rates of associated AEs in AA patients than W patients resulted in an overall greater rate of tabulated AEs in the category. Analogously, another group of drugs associated with significantly lower rates of AEs in most categories also moved up and down in frequency between the two racial categories and contributed to higher rates in overall tabulated AEs in AA patients than W patients. The combined effects of frequency of use and rate of induction of AEs accounted for the greater overall higher rates of AE in AA patients compared to W patients. The actual rates of associated AEs with a drug class varied within different stage groupings in the Outpatient and NCH datasets, further contributing to AE variation. However, the effect of the same drug was always higher in NCH groups than in Outpatient groups. These data suggest that there is a certain component of variability and uncertainty in assigning rates of AEs for specific drug categories within venues. However, global attribution of AEs is significantly greater in the NCH practice venue.

The vast majority of AE categories consisted of the same dozen or so AEs more than 98% of the time. There were conspicuous attributions of differences between the Outpatient and NCH settings for some of them. Several large differences in the frequency of some of the AEs based on race were also recorded. This further underscores the unique characteristics of venues and care providers in the two venues.

Our data suggest that treatment differences between patients of the two different races in the two venues are not based on the inherent frequencies of the patient groups but rather on the unique characteristics of the functioning models of the venues. It has been well documented that the use of chemotherapy treatments in the outpatient setting is higher than in the hospital setting [60], albeit the cost of hospital-setting care is greater than that in private practices [61]. A trend to integrate outpatient chemotherapy use into hospital-based settings by integrating practicing oncologists into those venues has decreased the use of chemotherapy drugs by these oncologists but shifted their use to more expensive treatments in hospital outpatient settings [60]. It is likely that follow-up and adherence to care are more efficient in the NCH setting, also contributing to the higher rates of treatment. A factor in this equation is certainly the continuity of care by a non-random-choice single caregiver in the NCH setting rather than a staff physician in the Outpatient setting [60].

To punctuate our findings, the rates of AEs in AA patients were globally higher than those in W patients, but the differences were small. However, we hypothesize that differences in AEs or perceived AEs by caregivers can result in differences in treatments that impact outcomes, as suggested previously [36]. It is likely that as more and more private oncology practices integrate into hospital-owned systems, the trends we observed in lesser treatment in the AA population in the NCH setting will begin to shift to the trend we see in the Outpatient database, where AA patients actually have higher rates of treatment and more equivalent rates of AEs. Treatment and perception biases regarding AEs and continuation of care in AA patients become less of a factor in more diverse hospital settings, to the benefit of dampening the trends observed in the NCH data. This analysis should guide investigations to further study this phenomenon. It should also prompt investigations into the impact of lower treatment rates of the same patient population in different venues. The impact of the different rates of treatment is not clear. Lower rates of completion of adjuvant chemotherapy may have an impact on recurrence, while less treatment of stage IV BC may affect survival. However, overtreatment of stage IV BC past the point of futility is also detrimental and decreases the quality of life without extending survival. Follow-up investigations need to determine the impacts of these differences on outcomes such as recurrence in the local disease setting or survival in the stage IV setting.

The molecular differences in cancer cells and the tumor microenvironment we noted earlier also need to be considered in the context of differences in AEs. While they certainly do not account for the differences we noted between the treatment venues, they need to be investigated with respect to significant differences in specific AEs in response to specific treatments. More importantly, the role of molecular differences, both genetic and epigenetic, need to be investigated in conjunction with large databases to account for differences in recurrence rates and treatment resistance.

## 5. Conclusions

This study demonstrates 1.3- to 2.8-fold higher treatment rates and 1.2- to 1.6-fold higher AEs in the NCH venues compared to the Outpatient venues that administer breast cancer treatment. Our data demonstrate that AA patients have lower cancer treatment rates in the NCH setting than W patients and suffer greater collective rates of AEs from cancer treatment in many categories. The differences in the rates of AEs arise from higher global rates of recorded AEs in the NCH setting and slightly higher rates of administration of drugs with higher associated AEs in AA patients than in W patients. Differences in patient age and comorbidity indices did not support their roles of the differences in AEs between the two racial groups. AEs were limited to a relatively short list of categories that comprised the vast majority of recorded AEs. We observed distinct differences in the rates of specific AE categories between the NCH setting and the Outpatient setting, suggesting caregiver and operational venue effects on the recording of AEs. We observed some distinct differences in the rates of some AEs between AA and W patients. These data suggest that caregiver or venue protocols likely affect treatment differences between venues, races, and recorded AEs. These differences may have an impact on differences in the completion or dosing of cancer treatment between the races.

## Figures and Tables

**Table 1:** Patient distributions by stage.

Stage	AA + W PTs	% in Stage	Outpatient					NCH				
			W	% in Stage	AA	% in Stage	AA/(W + AA) × 100%	W	% in Stage	AA	% in Stage	AA/(W + AA) × 100%
I	275,428	54.0	117,716	55.5	10,819	41.7	8.4	134,616	55.5	12,277	42.0	8.4
II	163,589	32.1	66,410	31.3	9949	38.3	13.0	76,118	31.4	11,112	38.0	12.7
III	53,486	10.5	21,202	10.0	3896	15.0	15.5	24,034	9.9	4354	14.9	15.3
IV	17,650	3.5	6947	3.3	1293	5.0	15.7	7942	3.3	1468	5.0	15.6
Total	510,153		212,275		25,957			242,710		29,211		

**Table 2:** Distribution of age and comorbidity indices by stage, race, and treatment setting.

	Outpatient				NCH			
	Age ± SD		Comorb. Index ± SD		Age + SD		Comorb. Index ± SD	
Stage	W	AA	W	AA	W	AA	W	AA
I–III	68.0 ± 10.8	64.6 ± 12.3 ^1^	4.2 ± 2.8	4.5 ± 2.9 ^1^	67.8 ± 10.8	64.6 ± 12.2 ^1^	3.6 ± 2.7 ^4^	4.0 ± 2.9 ^1,4^
I	68.4 + 10.0	66.0 + 10.9 ^1^	4.2 + 2.7	4.5 + 2.9 ^1^	68.2 + 10.1	66.0 + 10.8 ^1^	3.6 + 2.6 ^4^	4.1 + 2.8 ^1,4^
II	67.7 + 11.4	63.9 + 13.0 ^1^	4.2 + 2.8	4.5 + 3.0 ^1^	67.5 + 11.4	63.9 + 12.8 ^1^	3.7 + 2.7 ^4^	4.1 + 2.9 ^1,4^
III	66.5 + 12.2	62.2 + 13.9 ^1^	4.1 + 2.8	4.2 + 3.0 ^2^	66.4 + 12.2	62.3 + 13.8 ^1^	3.6 + 2.7 ^4^	3.9 + 2.9 ^1,4^
IV	69.4 + 12.1	65.3 + 13.3 ^1^	3.8 + 2.8	4.0 + 2.9 ^3^	69.5 + 12.0	65.6 + 13.1 ^1^	3.5 + 2.7 ^4^	3.7 + 2.8 ^1,4^

^1^ AA/W *p* = 0; ^2^ AA/W *p* = 0.05; ^3^ AA/W *p* = 0.022; ^4^ NCH/Outpatient *p* = 0 (*t*-test).

**Table 3:** Distribution of patient, cancer, treatment, and AE characteristics by race and venue.

	%	Age ± SD	Comb. Index ± SD	TR/PT	AE/TR	%	Age ± SD	Comb. Index ± SD	TR/PT	AE/TR
	W					AA				
**Stage I–III**										
**Outpatient**										
ER/PR+	84.6%	68.6 ± 10.5	4.2 ± 2.8	0.75	0.37	71.1% ^a^	65.9 ± 12.1 ^b^	4.5 ± 2.9 ^b^	1.40 ^a^	0.38
ER/PR-	15.4%	65.8 ± 11.4 ^d^	4.0 ± 2.8 ^d^	1.56 ^c^	0.40 ^c^	28.9% ^a^	61.8 ± 12.3 ^b,d^	4.2 ± 2.9 ^b,d^	2.45 ^a,c^	0.41 ^c^
Her2-	88.3%	71.8 ± 9.2	4.1 ± 2.8	0.92	0.40	85.3% ^a^	68.0 ± 11.4 ^b^	4.4 ± 3.0 ^b^	1.69 ^a^	0.41
Her2+	11.7%	70.2 ± 10.2 ^f^	4.0 ± 2.9 ^f^	4.31 ^e^	0.38 ^e^	14.7% ^a^	65.4 ± 12.1 ^b,f^	4.1 ± 3.0 ^f^	5.51 ^a,e^	0.38 ^e^
**NCH**										
ER/PR+	84.6%	68.4 ± 10.5 ^h^	3.6 ± 2.7 ^h^	1.96 ^g^	0.52 ^g^	71.2% ^a^	65.9 ± 11.9 ^b^	4.1 ± 2.9 ^b,h^	1.70 ^a,g^	0.56 ^a,g^
ER/PR-	15.4%	65.6 ± 11.4 ^d,h^	3.5 ± 2.6 ^d,h^	3.84 ^c,g^	0.57 ^c,g^	28.8% ^a^	61.9 ± 12.2 ^b,d^	3.9 ± 2.8 ^b,d,h^	2.85 ^a,c,g^	0.57 ^g^
Her2-	88.4%	71.7 ± 9.1	3.6 ± 2.7 ^h^	1.54 ^g^	0.50 ^g^	85.5% ^a^	68.0 ± 11.2 ^b^	4.0 ± 3.0 ^b,h^	1.37 ^a,g^	0.53 ^a,g^
Her2+	11.6%	70.2 ± 10.0 ^f^	3.5 ± 2.8 ^f,h^	6.57 ^e,g^	0.49 ^g^	14.5% ^a^	65.7 ± 11.8 ^b,f^	3.8 ± 2.9 ^b,f,h^	3.70 ^a,e,g^	0.45 ^a,e,g^
**Stage IV**										
**Outpatient**										
ER/PR+	81.6%	69.3 ± 12.0	3.8 ± 2.8	5.09	0.34	72.3% ^a^	65.1 ± 13.6 ^b^	3.9 ± 2.9	6.08 ^a^	0.40 ^a^
ER/PR-	18.4%	68.6 ± 12.3	3.8 ± 3.0	5.48 ^c^	0.35	27.7% ^a^	65.2 ± 13.1 ^b^	4.2 ± 3.1 ^b^	6.85 ^a^	0.34 ^c^
Her2-	77.3%	71.4 ± 11.0	4.0 ± 2.9	4.43	0.38	73.7% ^a^	68.0 ± 12.2 ^b^	4.3 ± 3.1 ^b^	4.55	0.42 ^a^
Her2+	22.7%	68.3 ± 12.4 ^f^	3.6 ± 2.9 ^f^	8.52 ^e^	0.28 ^e^	26.3% ^a^	63.4 ± 14.0 ^b,f^	3.5 ± 2.7 ^f^	8.38 ^e^	0.29 ^e^
**NCH**										
ER/PR+	81.6%	69.5 ± 11.9	3.4 ± 2.7 ^h^	9.79 ^g^	0.56 ^g^	72.7% ^a^	65.4 ± 13.3	3.5 ± 2.8 ^h^	5.31 ^a,g^	0.52 ^a,g^
ER/PR-	18.4%	69.0 ± 12.1	3.5 ± 2.8 ^h^	10.92 ^c,g^	0.55 ^g^	27.3% ^a^	65.6 ± 12.8 ^b^	3.9 ± 2.9 ^b,d^	6.43 ^a,c^	0.54 ^g^
Her2-	77.9%	71.6 ± 10.9	3.5 ± 2.8 ^h^	4.08 ^g^	0.53 ^g^	74.4% ^a^	68.1 ± 11.9 ^b^	3.9 ± 3.0 ^b,h^	3.85 ^g^	0.53 ^g^
Her2+	22.1%	68.7 ± 12.2 ^f^	3.3 ± 2.8 ^h^	13.09 ^e,g^	0.52 ^g^	25.6% ^a^	64.0 ± 13.8 ^b,f,h^	3.4 ± 2.7	3.29 ^a,g^	0.49 ^g^

**Legend.** Statistically significant difference between AA and W patients by ^a^ Chi-square or ^b^ *t*-test; ER/PR+ and ER/PR- by ^c^ Chi-square or ^d^ *t*-test; Her2- and Her2+ by ^e^ Chi-square or ^f^ *t*-test. Outpatient and NCH by ^g^ Chi-square or ^h^ *t*-test.

**Table 4:** Most frequent treatments in stage I–III Outpatient and NCH patients.

Stage I–III						
Treatment	Patient TRs	% of All TRs	% TRs with AEs	% AEs/TR	Age ± SD	Comorbidity Index ± SD
**Outpatient**						
Taxanes	54,178	24.1%	38.3%	9.2%	63.8 ± 10.5	3.9 ± 2.5
Her2 targeting agents	41,037	18.2%	36.4%	6.6%	64.5 ± 11.2	4.0 ± 2.6
Bisphosphonates	25,976	11.5%	30.0%	3.5%	65.5 ± 11.1	4.3 ± 2.6
Alkylating agents	23,921	10.6%	29.1%	3.1%	65.5 ± 9.5	4.2 ± 2.5
Anthracyclines	17,380	7.7%	54.2%	4.2%	63.9 ± 9.4	4.0 ± 2.3
Pyrimidine analogs	13,960	6.2%	42.7%	2.7%	61.9 ± 12.2	4.1 ± 2.6
Antiestrogens	13,723	6.1%	27.8%	1.7%	63.3 ± 12.5	4.4 ± 2.7
Platinum cmpds.	13,557	6.0%	55.6%	3.4%	62.7 ± 11.0	3.9 ± 2.4
Vinca alkaloids	6427	2.9%	36.0%	1.0%	57.5 ± 12.6	3.5 ± 2.5
Folate antagonists	5325	2.4%	23.7%	0.6%	66.3 ± 10.8	4.7 ± 2.6
VEGF inhibitors	3696	1.6%	37.9%	0.6%	60.7 ± 13.2	3.8 ± 2.5
Non chemother.	2781	1.2%	41.3%	0.5%	67.2 ± 11.3	5.3 ± 2.8
Total tallied	221,961	98.7%		37.0%	63.6 ± 11.3	4.2 ± 2.6
**NCH**						
Taxanes	130,739	21.2%	57.0%	12.1%	65.6 ± 9.6	4.0 ± 2.4
Her2 targeting agents	98,222	15.9%	51.7%	8.2%	66.4 ± 10.4	4.0 ± 2.5
Anthracyclines	63,847	10.4%	72.5%	7.5%	66.1 ± 8.5	4.2 ± 2.3
Pyrimidine analogs	57,783	9.4%	66.5%	6.2%	65.9 ± 10.6	4.5 ± 2.5
Bisphosphonates	55,761	9.1%	35.1%	3.2%	67.1 ± 10.5	4.4 ± 2.5
VEGF inhibitors	49,015	8.0%	29.5%	2.3%	71.2 ± 11.1	5.2 ± 2.5
Alkylating agents	47,241	7.7%	51.8%	4.0%	67.0 ± 8.6	4.1 ± 2.4
Platinum cmpds.	35,885	5.8%	67.7%	3.9%	64.8 ± 10.2	4.0 ± 2.4
Antiestrogens	25,883	4.2%	37.5%	1.6%	65.4 ± 11.5	4.4 ± 2.5
Folate antagonists	23,502	3.8%	57.9%	2.2%	68.9 ± 8.9	5.1 ± 2.4
Vinca alkaloids	22,480	3.7%	55.3%	2.0%	61.5 ± 12.4	3.8 ± 2.4
Total tallied	610,358	98.1%		53.3%	66.4 ± 10.2	4.3 ± 2.4
Significance Outpatient/NCH *t*-test					*p* = 0	*p* = 0

**Table 5:** Most frequent treatments in stage IV Outpatient and NCH patients.

Stage IV						
Treatment	Patient TRs	% of all TRs	% TRs with AEs	% AEs/TR	Age ± SD	Comorbidity Index ± SD
**Outpatient**						
Taxanes	9687	22.0%	33.5%	7.4%	63.1 ± 11.8	3.3 ± 2.5
Her2 targeting agents	8729	19.8%	31.4%	6.2%	62.2 ± 12.5	3.2 ± 2.7
Bisphosphonates	8559	19.4%	30.6%	5.9%	65.5 ± 12.0	3.6 ± 2.7
Antiestrogens	4897	11.1%	21.1%	2.3%	67.1 ± 11.0	3.8 ± 2.7
Pyrimidine analogs	3077	7.0%	39.1%	2.7%	62.1 ± 11.8	3.4 ± 2.6
Vinca alkaloids	2179	4.9%	32.9%	1.6%	61.0 ± 11.6	3.2 ± 2.6
Platinum cmpds.	1760	4.0%	54.8%	2.2%	61.4 ± 12.3	3.1 ± 2.4
Anthracyclines	1374	3.1%	50.5%	1.6%	62.1 ± 11.1	3.3 ± 2.5
Alkylating agents	1350	3.1%	30.1%	0.9%	63.0 ± 11.4	3.5 ± 2.6
VEGF inhibitors	1094	2.5%	37.0%	0.9%	61.3 ± 11.1	2.7 ± 2.1
Her2 ab + DM1 antitubulin	593	1.3%	25.8%	0.3%	58.9 ± 13.1	2.8 ± 2.7
Total tallied	43,299	98.2%		32.1%	62.5 ± 11.8	3.3 ± 2.5
**NCH**						
Taxanes	21,747	23.9%	52.6%	12.6%	65.5 ± 10.6	3.6 + 2.5
Her2 targeting agents	18,898	20.7%	52.9%	11.0%	64.7 + 11.9	3.4 ± 2.5
Bisphosphonates	12,422	13.6%	54.2%	7.4%	66.9 ± 11.5	3.8 ± 2.6
Pyrimidine analogs	8856	9.7%	62.8%	6.1%	64.4 ± 11.1	3.6 ± 2.5
Antiestrogens	5879	6.5%	38.9%	2.5%	68.1 ± 11.3	3.9 ± 2.6
Vinca alkaloids	5264	5.8%	55.1%	3.2%	64.2 ± 12.1	3.5 ± 2.4
Platinum cmpds.	4626	5.1%	69.4%	3.5%	64.8 ± 10.6	3.4 ± 2.4
VEGF inhibitors	4613	5.1%	53.8%	2.7%	66.6 ± 12.0	3.9 ± 2.6
Anthracyclines	3054	3.4%	69.9%	2.3%	65.1 ± 10.2	3.5 ± 2.2
Alkylating agents	2737	3.0%	57.4%	1.7%	65.5 ± 10.0	3.5 ± 2.5
Folate analogs	2116	2.3%	64.6%	1.5%	65.7 ± 11.0	4.0 ± 2.8
Total tallied	90,212	99.0%		54.5%	65.6 ± 11.1	3.7 ± 2.5
Significance Outpatient/NCH *t*-test					*p* = 0	*p* = 0

**Table 6:** Most frequent AEs in stage I–III and stage IV Outpatient and NCH patients.

AEs	AEs with TRs	% of all TR AEs	AEs	AEs with TRs	% of all TR AEs
Outpatient			NCH		
**Stage I–III**					
Nausea/vomiting	22,058	26.1%	Nausea/vomiting	176,989	53.4%
Weakness/malaise, delirium	19,781	23.4%	Electrolyte abnormalities	30,892	9.3%
Neutropenia/Leukopenia	13,273	15.7%	Weakness/malaise, delirium	29,611	8.9%
Respiratory symptoms.	6470	7.7%	Neutropenia/Leukopenia	29,329	8.8%
Anemias	4518	5.4%	Anemias	26,208	7.9%
Infection/fever	4185	5.0%	Respiratory symptoms	11,771	3.5%
Electrolyte abnormalities	3814	4.5%	Infection/fever	10,400	3.1%
Diarrhea	2882	3.4%	Thrombophilia	5647	1.7%
Thrombophilia	2531	3.0%	Diarrhea	3242	1.0%
Constipation	2214	2.6%			
Pulm. Embolus	1163	1.4%			
Weight loss/malnutrition	859	1.0%			
Total tallied	83,748	99.2%	Total tallied	324,089	97.7%
**Stage IV**					
Weakness/malaise, delirium	3731	24.5%	Nausea/vomiting	22,939	45.7%
Nausea/vomiting	3342	22.0%	Electrolyte abnormalities	6492	12.9%
Neutropenia/Leukopenia	1828	12.0%	Weakness/malaise, delirium	5994	11.9%
Respiratory symptoms	1412	9.3%	Anemias	4588	9.1%
Anemias	1095	7.2%	Neutropenia/Leukopenia	3588	7.2%
Infection/fever	850	5.6%	Respiratory symptoms	2497	5.0%
Thrombophilia	801	5.3%	Infection/fever	1154	2.3%
Electrolyte abnormalities	627	4.1%	Thrombophilia	1047	2.1%
Constipation	461	3.0%	Pulmonary Embolus	623	1.2%
Diarrhea	410	2.7%	Diarrhea	536	1.1%
Pulmonary Embolus	407	2.7%			
Weight loss/malnutrition	156	1.0%			
Total tallied	15,120	99.5%	Total tallied	49,458	98.6%

**Table 7:** (**A**) Race, age, comorbidity index, and venue distribution of treatments and AEs in stage I–III. (**B**) Race, age, comorbidity index, and venue distribution of treatments and AEs in stage IV.

**(A)**										
**Stage I–III**										
**Treatment**	**Patient TRs**	**% of All TRs**	**% AEs/TR**	**Age ± SD**	**Comorb.** **Ind. ± SD**	**Patient TRs**	**% of All TRs**	**% AEs/TR**	**Age ± SD**	**Comorb.** **Ind. ± SD**
**Outpatient**						**NCH**				
**White patients**										
Taxanes	40,541	23.6%	9.1%	64.7 ± 10.0	3.8 ± 2.5	109,345	20.9%	11.9%	66.1 ± 9.2	4.0 ± 2.4
Her2 target. agts	31,093	18.1%	6.7%	65.4 ± 10.8	3.9 ± 2.6	82,328	15.7%	8.1%	66.9 ± 10.1	4.0 ± 2.5
Bisphosphonates	21,771	12.6%	3.7%	66.1 ± 10.5	4.3 ± 2.6	49,594	9.5%	3.3%	67.3 ± 10.4	4.4 ± 2.5
Alkylating agents	18,036	10.5%	3.0%	66.3 ± 9.0	4.1 ± 2.5	39,253	7.5%	3.9%	67.4 ± 8.2	4.0 ± 2.4
Anthracyclines	12,865	7.5%	4.0%	64.8 ± 8.7	3.9 ± 2.2	52,960	10.1%	7.3%	66.5 ± 8.2	4.2 ± 2.3
Antiestrogens	11,392	6.6%	1.9%	64.1 ± 12.1	4.4 ± 2.7	23,246	4.4%	1.7%	65.8 ± 11.2	4.4 ± 2.4
Pyrimidine anlgs.	10,120	5.9%	2.6%	63.1 ± 11.5	4.1 ± 2.6	48,948	9.3%	6.2%	66.4 ± 10.2	4.5 ± 2.5
Platinum cmpds.	9835	5.7%	3.2%	63.8 ± 10.2	3.8 ± 2.4	29,312	5.6%	3.8%	65.3 ± 9.8	4.0 ± 2.4
Vinca alkaloids	4958	2.9%	1.0%	58.5 ± 12.6	3.6 ± 2.5	18,548	3.5%	1.9%	62.1 ± 12.2	3.8 ± 2.4
Folate analogs	4296	2.5%	0.6%	66.9 ± 10.2	4.6 ± 2.6	20,416	3.9%	2.3%	69.2 ± 8.6	5.0 ± 2.4
VEGF inhibitors	2706	1.6%	0.6%	62.1 ± 12.8	3.9 ± 2.5	45,219	8.6%	3.4%	71.9 ± 10.8	5.2 ± 2.5
Non chemother.	2284	1.3%	0.5%	67.7 ± 11.0	5.1 ± 2.8					
Total tallied	169,897	98.7%	36.9%	64.5 ± 10.8	4.1 ± 2.5	519,169	99.1%	53.7%	66.8 ± 9.9	4.3 ± 2.4
**African American Patients**										
Taxanes	10,768	26.3%	10.0%	60.2 ± 11.6	4.3 ± 2.6	13.603	23.9%	13.2%	61.7 ± 11.3	4.4 ± 2.6
Her2 target. agts.	7551	18.4%	6.8%	60.7 ± 12.0	4.3 ± 2.7	8.750	15.4%	7.8%	62.0 ± 12.1	4.2 ± 2.5
Alkylating agents	4772	11.7%	3.5%	62.1 ± 10.5	4.6 ± 2.6	5366	9.4%	4.7%	63.6 ± 10.3	4.5 ± 2.6
Anthracyclines	3794	9.3%	5.0%	60.8 ± 10.7	4.5 ± 2.5	7947	13.9%	10.1%	62.9 ± 9.6	4.7 ± 2.5
Pyrimidine anlgs.	3062	7.5%	2.9%	57.4 ± 13.1	4.1 ± 2.6	5524	9.7%	6.2%	63.0 ± 12.2	4.9 ± 2.6
Platinum cmpds.	3044	7.4%	4.3%	58.5 ± 12.3	4.1 ± 2.5	3852	6.8%	4.4%	60.5 ± 12.0	4.4 ± 2.6
Bisphosphonates	2691	6.6%	2.3%	59.6 ± 13.8	4.2 ± 2.8	3012	5.3%	2.5%	63.3 ± 12.2	4.7 ± 2.8
Antiestrogens	1678	4.1%	1.1%	59.2 ± 13.9	4.4 ± 2.7	1320	2.3%	0.9%	61.1 ± 14.1	4.6 ± 2.7
Vinca alkaloids	1122	2.7%	1.0%	53.1 ± 12.0	3.3 ± 2.4	2829	5.0%	2.8%	57.5 ± 13.6	3.7 ± 2.5
VEGF inhibitors	783	1.9%	0.8%	55.0 ± 13.0	3.8 ± 2.6	2023	3.6%	1.4%	64.2 ± 12.4	5.5 ± 2.8
Folate analogs	755	1.8%	0.4%	62.8 ± 12.8	5.1 ± 2.7	2047	3.6%	2.1%	66.6 ± 10.8	5.8 ± 2.5
Total tallied	40,020	97.8%	38.1%	59.0 ± 12.3	4.3 ± 2.6	56,273	98.8%	56.0%	62.4 ± 11.9	4.7 ± 2.6
Significance W/AA *t*-test				*p* = 0	*p* = 0				*p* = 0	*p* = 0
**(B)**										
**Stage IV**										
**Treatment**	**Patient TRs**	**% of all TRs**	**% AEs/TR**	**Age ± SD**	**Comorb.** **Ind. ± SD**	**Patient TRs**	**% of All TRs**	**% AEs/TR**	**Age ± SD**	**Comorb.** **Ind. ± SD**
**Outpatient**						**NCH**				
**White patients**										
Taxanes	7229	21.0%	6.8%	64.0 ± 11.4	3.3 ± 2.5	18,954	24.1%	12.7%	65.9 ± 10.2	3.6 ± 2.4
Bisphosphonates	7001	20.3%	8.0%	66.4 ± 11.6	3.7 ± 2.7	10,674	13.5%	7.4%	67.7 ± 11.2	3.9 ± 2.6
Her2 target. agts.	6653	19.3%	6.2%	63.4 ± 12.1	3.3 ± 2.8	16,209	20.6%	10.9%	65.4 ± 11.8	3.4 ± 2.6
Antiestrogens	4113	11.9%	2.3%	67.8 ± 10.8	3.8 ± 2.7	5157	6.5%	2.5%	68.6 ± 11.1	4.0 ± 2.6
Pyrimidine anlgs.	2445	7.1%	2.9%	63.3 ± 11.5	3.5 ± 2.6	7867	10.0%	6.4%	64.9 ± 10.6	3.6 ± 2.5
Vinca alkaloids	1712	5.0%	1.6%	61.7 ± 11.5	3.3 ± 2.6	4532	5.8%	3.2%	64.6 ± 11.6	3.6 ± 2.4
Platinum cmpds.	1378	4.0%	2.2%	62.3 ± 11.4	3.1 ± 2.3	3688	4.7%	3.2%	65.6 ± 9.9	3.5 ± 2.5
Anthracyclines	1055	3.1%	1.6%	63.5 ± 10.4	3.2 ± 2.4	2649	3.4%	2.4%	65.4 ± 9.9	3.5 ± 2.2
Alkylating agents	1013	2.9%	0.8%	64.1 ± 11.0	3.4 ± 2.1	2364	3.0%	1.7%	65.4 ± 9.7	3.5 ± 2.5
VEGF inhibitors	799	2.3%	0.8%	62.0 ± 10.9	2.8 ± 2.1	3939	5.0%	2.6%	67.2 ± 11.6	3.9 ± 2.6
Her2 ab+DM1	386	1.1%	0.3%	61.5 ± 12.8	3.1 ± 3.1	0	0	0	0	0
Folate analogs	341	1.0%	0.3%	63.5 ± 12.2	3.6 ± 2.7	2062	2.6%	1.7%	65.6 ± 10.5	4.0 ± 2.9
Total tallied	34,125	99.0%	33.8%	63.6 ± 11.4	3.3 ± 2.6	78,095	99.1%	54.7%	66.0 ± 10.7	3.7 ± 2.5
**African American Patients**										
Taxanes	2052	27.4%	10.1%	60.0 ± 12.7	3.7 ± 2.7	1933	24.4%	12.0%	63.1 ± 12.9	4.0 ± 2.8
Her2 target. agts.	1483	19.8%	6.6%	57.0 ± 13.7	3.2 ± 2.3	1313	16.6%	8.2%	60.3 ± 12.2	3.1 ± 2.2
Bisphosphonates	1174	15.7%	6.4%	61.3 ± 13.1	3.5 ± 2.7	1117	14.1%	7.3%	62.3 ± 12.4	4.1 ± 2.6
Antiestrogens	585	7.8%	2.7%	63.5 ± 12.0	3.9 ± 2.7	494	6.2%	2.9%	63.1 ± 13.7	4.0 ± 2.6
Pyrimidine anlgs.	445	5.9%	2.0%	58.6 ± 12.2	3.5 ± 2.7	709	8.9%	4.7%	60.5 ± 13.6	3.2 ± 2.5
Vinca alkaloids	374	5.0%	1.7%	59.6 ± 11.6	3.1 ± 2.5	571	7.2%	3.8%	59.0 ± 15.3	3.0 ± 2.3
Platinum cmpds.	310	4.1%	2.3%	59.2 ± 14.0	3.8 ± 2.8	783	9.9%	6.8%	61.2 ± 12.8	3.3 ± 2.3
Anthracyclines	267	3.6%	1.5%	58.5 ± 11.8	3.7 ± 2.8	298	3.8%	2.4%	64.5 ± 10.2	3.6 ± 2.3
VEGF inhibitors	260	3.5%	2.1%	57.4 ± 12.8	2.6 ± 2.2	286	3.6%	1.9%	58.9 ± 13.9	3.0 ± 1.9
Alkylating agents	257	3.4%	1.1%	59.4 ± 11.6	4.2 ± 3.1	260	3.3%	1.6%	66.7 ± 11.2	3.7 ± 2.4
Her2 ab + DM1	170	2.3%	0.7%	50.0 ± 11.4	2.5 ± 1.8	0	0	0	0	0
Total tallied	7377	98.6%	37.1%	58.6 ± 12.5	3.4 ± 2.6	7764	97.9%	51.8%	62.0 ± 12.8	3.5 ± 2.4
Significance W/AA *t*-test				*p* = 0	*p* = 0.003				*p* = 0	*p* = 0

## Data Availability

Original data were obtained from SEER-Medicare under a two-tiered review process.

## Supplementary Materials

The following are available online at https://www.mdpi.com/2072-6694/15/17/4333/s1, Table S1: Distribution of patient characteristics, tumor markers, treatments, and AEs in stage I–III Outpatient dataset patients; Table S2: Distribution of patient characteristics, tumor markers, treatments, and AEs in stage I–III NCH dataset patients; Table S3: Distribution of patient characteristics, tumor markers, treatments. and AEs in stage IV Outpatient dataset patients; Table S4: Distribution of patient characteristics, tumor markers, treatments, and AEs in stage IV NCH dataset patients; Table S5: Treatments associated with common AE differences in stage I–III Outpatient vs. NCH; Table S6: Treatments associated with common AE differences in stage IV Outpatient v. NCH sets; Table S7: Race distribution of the most frequent AEs in stage I–III Outpatient and NCH patients; Table S8: Race distribution of the most frequent AEs in stage IV Outpatient and NCH patients.

## Appendix A

We obtained the data we analyzed in this manuscript from the National Cancer Institute (NCI) SEER-Medicare (S-M) program under dual review with the understanding that S-M must review and approve publications using their data. We unintentionally overlooked having the manuscript reviewed by NCI before its publication. After guidance and review by S-M, we generated new, more specific subsets of the datasets defining institutional outpatient care and private practice office care venues and we confirmed that the additional classifications had no impact on the conclusions of the study. Although the inferences were ultimately similar between the published analysis and this analysis, this addendum was necessary because the NCH file cannot be classified correctly as a distinct service venue. The NCH file contains claims from all types of physician services (and other non-institutional providers), regardless of whether these services were provided in inpatient/outpatient hospital settings or in physicians’ offices. In this addendum, we clarify some of the datasets we used to generate patient treatment (TR) and adverse event (AE) relationships between institutional outpatient care providers and private practice office care providers. Nothwithstanding, our inferences comparing the S-M Outpatient and NCH datasets in the published manuscript are valid.

To address the question of different venues of care, we used the data from the NCH and Outpatient datasets to generate two new data subsets, identified as the Institutional Outpatient (Inst. OP) and Private Practice (PP) Office datasets, to analyze differences in patients, care, and AEs in these two outpatient venues. We restricted our study population, in addition to the prior eligibility criteria we used, to patients with Medicare A and B, no MC HMO, and age at the time of enrollment ≥ 65 years, enrolled for age not for disability.

We regenerated our data according to the following inclusion criteria: claims whose CLM_TYPE = [40 Outpt, 41 Outpt full encounter, 42 Outpt abbreviated, and 71 RUIC O local carrier non-DMEPOS claim] whose OPSRVTYP = 3 are included for both NCH and Outpatient datasets. A typical NCH file has plcsrvc_nch = [11 Ofc] (Private Practice Office care provider) and plcsrvc_outp = [13 Assisted Living, 22 Outpatient Hospital, 26 Military Treatment Facility, 31 Skilled Nursing Facility, 32 Nursing Facility, 50 Federally Qualified Health Center, 71 State and Local Public Health Clinic, 72 Rural Health Clinic]. A typical Outpatient file has opsrvtype = [3 Institutional Outpatient providers], fac_type_outpt = [1 Hosp, and 2 Skilled Nursing Facility]. This approach was approved by S-M review.

In the 488 NCH files there were 162,772,263 records. Of these, 93,587,805 records (57.5%) were Private Practice Office records and 18,904,104 records (11.6%) were Institutional Outpatient records. In the 380 Outpatient files, there were 75,218,148 records. Of these, 0 were Private Practice Office records and 57,990,140 records (77.1%) were Institutional Outpatient records. Thus, 75.4% of Inst. OP records originated from the Outpatient files and 24.6% originated from the NCH files, whereas 100% of the PP Office records originated from the NCH files.

The distributions of patients in the Inst. OP dataset were 88.0 W, 7.3 AA and 4.7% other races, and in the PP Office dataset, they were 87.9 W, 7.2 AA and 4.9% other races (Table A1), similar to the Outpatient and NCH dataset values. Most patients had stage I BC in both datasets, with progressively smaller percentages with advancing stages. The distributions of patterns were significantly different between W and AA patients, with AA patients having far fewer early-stage BC cases and higher rates of later-stage BC than W patients in both Inst. OP and PP Office datasets. This trend was analogous to the Outpatient and NCH data, although there were small differences in absolute values.

**Table A1:** Patient distribution by stage.

	Inst. Outpt.							PP Office						
Stage	All	% in Stage	W	% in Stage	AA	% in Stage	AA/ (W+AA × 100%)	All	% in Stage	W	% in Stage	AA	% in Stage	AA/ (W+AA × 100%)
I	125,391	53.1%	112,023	53.9%	7292	42.1%	6.1%	129,105	53.2%	115,191	54.0%	7345	42.3%	6.0%
II	71,862	30.4%	62,544	30.1%	5890	34.0%	8.6%	73,742	30.4%	64,143	30.1%	5924	34.1%	8.5%
III	25,998	11.0%	22,203	10.7%	2675	15.4%	10.8%	26,603	11.0%	22,758	10.7%	2661	15.3%	10.5%
IV	12,947	5.5%	11,021	5.3%	1483	8.6%	11.9%	13,148	5.4%	11,261	5.3%	1431	8.2%	11.3%
Total	236,198		207,791		17,340			242,598		213,353		17,361		

We compared the differences in the distributions of age, comorbidity index, and treatment and AE rates by stage grouping and race between the Inst. OP and the PP Office settings to the differences between the Outpatient and NCH datasets for statistical significance (Table A2). We counted individual treatments multiple times, each time a 0, 1, 2, ..., n AE was recorded. The data confirmed that the age distributions of the Inst. OP and PP Ofc. datasets were higher than in the Outpatient/NCH datasets, corresponding to the inclusion of only patients who qualified by age ≥ 65 years. Overall, the data showed concurrences between the two comparisons in the effects of ER/PR (), Her2 () status, and race () on distribution differences in age, comorbidity index, TR/PT, and AE/TR. The congruence between the Outpatient/NCH and Inst. OP/PP Ofc. datasets () was most evident in files that were annotated for ER/PR status and less so in the files annotated for Her2 that had much fewer numbers.

**Table A2:** Distribution of age, comorbidity index, and treatment and AE rates by stage group race and treatment setting.

	W					AA				
	% ER/PR-	Age	Comb	TR/PT	AE/TR	% ER/PR-	Age	Comb	TR/PT	AE/TR
**Stage I–III**										
**Inst. Outpt.**										
ER/PR+	85.8	75.3 ± 7.2	3.0 ± 3.1	0.6	0.40	75.8 ^a^	75.0 ± 7.2 ^b^	3.5 ± 3.3 ^b^	0.9 ^a^	0.39
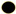
ER/PR-	14.2	75.0 ± 7.3 ^d^	2.7 ± 3.1 ^d^	1.5 ^c^	0.43 ^c^	24.4 ^a^	74.0 ± 7.0 ^b,d^	3.1 ± 3.3 ^b,d^	2.2 ^a,c^	0.43 ^c^
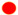
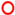
Her2-	89.3	75.0 ± 7.3	3.3 ± 3.0	0.9	0.42	87.4 ^a^	74.6 ± 7.3 ^b^	3.8 ± 3.2 ^b^	1.3 ^a^	0.43
Her2+	10.7	74.7 ± 7.5 ^f^	3.4 ± 3.1 ^f^	4.7 ^e^	0.40 ^e^	12.6 ^a^	74.3 ± 7.1	3.6 ± 3.1	5.5 ^a,e^	0.36 ^a,e^
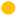
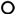
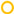
**PP Office**										
ER/PR+	85.8	75.3 ± 7.2	2.7 ± 2.8 ^h^	1.8 ^g^	0.52 ^g^	75.7 ^a^	75.0 ± 7.2 ^b^	3.1 ± 3.0 ^b,h^	1.6 ^a,g^	0.58 ^a,g^
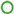
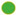
ER/PR-	14.2	75.0 ± 7.2 ^d^	2.4 ± 2.7 ^d,h^	4.2 ^c,g^	0.60 ^c,g^	24.3 ^a^	74.0 ± 6.9 ^b,d^	2.8 ± 3.0 ^b,d,h^	3.4 ^a,c,g^	0.59 ^g^
Her2-	89.3	74.9 ± 7.3 ^h^	3.1 ± 2.8 ^h^	1.6 ^g^	0.48 ^g^	87.5 ^a^	74.6 ± 7.2 ^b^	3.6 ± 3.0 ^b,h^	1.4 ^a^	0.52 ^a,g^
Her2+	10.7	74.5 ± 7.5 ^f^	3.1 ± 2.8 ^h^	7.3 ^e,g^	0.49 ^e,g^	12.5 ^a^	74.3 ± 7.1	3.4 ± 2.9	4.5 ^a,e,g^	0.46 ^a,e,g^
**Stage IV**										
**Inst. Outpt.**										
ER/PR+	79.4	76.1 ± 7.5	2.0 ± 2.8	3.0	0.37	68.0 ^a^	75.1 ± 7.4 ^b^	2.1 ± 2.9	2.9	0.39
ER/PR-	20.6	75.8 ± 7.5	1.7 ± 2.8 ^d^	3.0	0.37	32.0 ^a^	74.6 ± 6.8 ^b^	1.8 ± 2.9	2.8	0.42 ^a^
Her2-	80.6	75.9 ± 7.8	3.1 ± 3.0	4.2	0.40	79.0	75.0 ± 7.6 ^b^	3.2 ± 3.1	3.9	0.42
Her2+	19.4	75.3 ± 7.7	3.1 ± 3.1	8.4 ^e^	0.32 ^e^	21.0	74.6 ± 7.6	3.1 ± 3.2	4.0 ^a^	0.25 ^a,e^
**PP Office**										
ER/PR+	79.2	76.2 ± 7.5	1.8 ± 2.6 ^h^	6.4 ^g^	0.57 ^g^	68.1 ^a^	75.2 ± 7.4 ^b^	2.0 ± 2.7	4.7 ^a,g^	0.59 ^g^
ER/PR-	20.8	75.9 ± 7.5	1.6 ± 2.6 ^d^	7.7 ^c,g^	0.6 ^c,g^	31.9 ^a^	75.2 ± 6.8	1.6 ± 2.7 ^d^	4.3 ^a,g^	0.64 ^g^
Her2-	80.4	76.0 ± 7.8	2.8 ± 2.8 ^h^	3.9 ^g^	0.51 ^g^	78.7	75.2 ± 7.5	3.1 ± 2.9	3.9	0.55 ^g^
Her2+	19.6	75.3 ± 7.7 ^f^	2.8 ± 2.9	12.8 ^e,g^	0.54 ^e,g^	21.3	74.8 ± 7.8	2.8 ± 2.8	3.3 ^a^	0.38 ^a,e,g^

**Legend.** Statistically significant difference between: AA and W patients by ^a^ Chi-square or ^b^ *t*-test; ER/PR+ and ER/PR- by ^c^ Chi-square or ^d^ *t*-test; Her2- and Her2+ by ^e^ Chi-square or ^f^ *t*-test; Inst. Outpatient and PP Office by ^g^ Chi-square or ^h^ *t*-test. Correlations between Outpatient file and Institutional Outpatient data and between NCH file and Private Practice Office data. Correlation of the race effect (Yes /No ), ER/PR effect (Yes /No ), Her2 effect (Yes /No ), and venue effect (Yes /No ).

Overall, the data showed congruity between the general conclusions derived from comparing the Outpatient to the NCH and Inst. OP to the PP Ofc. datasets, with some exceptions where the number of entries was relatively small. The most striking conclusions, however, were the agreements between the Outpatient and NCH patterns compared with the Inst. OP and the PP Ofc. patterns in the four values of age, comorbidity index, TR/PT, and AE/TR in both W and AA patients designated by the green solid dots. Some of the stage IV categories that had more variability and fewer entries were less congruent than the stage I–III categories. We did not compare the adverse event categories in the two datasets for statistical differences. They remained similar in distribution, which varied slightly according to race, as in the primary study.
