# Correction: Wieder, R.; Adam, N. Racial Disparities in Breast Cancer Treatments and Adverse Events in the SEER-Medicare Data. *Cancers* 2023, *15*, 4333

**DOI:** 10.3390/cancers16132292

**Published:** 2024-06-21

**Authors:** Robert Wieder, Nabil Adam

**Affiliations:** 1 Rutgers New Jersey Medical School and the Cancer Institute of New Jersey, 185 South Orange Avenue, MSB F671, Newark, NJ 07103, USA; 2 Phalcon, LLC, Manhasset, NY 11030, USA; n.adam@phalconllc.com

## Newly Added Appendix A and Acknowledgements

We obtained the data we analyzed in this manuscript [1] from the National Cancer Institute (NCI) SEER-Medicare (S-M) program under dual review with the understanding that S-M must review and approve publications using their data. We unintentionally overlooked having the manuscript reviewed by NCI before its publication. This Appendix A was necessary because the NCH file cannot be classified correctly as a distinct service venue. After guidance and review by S-M, we generated new, more specific subsets of the datasets defining Institutional Outpatient care and Private Practice Office care venues and we confirmed that the additional classifications had no impact on the conclusions and inferences of the study.

**Appendix A**

We obtained the data we analyzed in this manuscript from the National Cancer Institute (NCI) SEER-Medicare (S-M) program under dual review with the understanding that S-M must review and approve publications using their data. We unintentionally overlooked having the manuscript reviewed by NCI before its publication. After guidance and review by S-M, we generated new, more specific subsets of the datasets defining institutional outpatient care and private practice office care venues and we confirmed that the additional classifications had no impact on the conclusions of the study. Although the inferences were ultimately similar between the published analysis and this analysis, this addendum was necessary because the NCH file cannot be classified correctly as a distinct service venue. The NCH file contains claims from all types of physician services (and other non-institutional providers), regardless of whether these services were provided in inpatient/outpatient hospital settings or in physicians’ offices. In this addendum, we clarify some of the datasets we used to generate patient treatment (TR) and adverse event (AE) relationships between institutional outpatient care providers and private practice office care providers. Nothwithstanding, our inferences comparing the S-M Outpatient and NCH datasets in the published manuscript are valid. 

To address the question of different venues of care, we used the data from the NCH and Outpatient datasets to generate two new data subsets, identified as the Institutional Outpatient (Inst. OP) and Private Practice (PP) Office datasets, to analyze differences in patients, care, and AEs in these two outpatient venues. We restricted our study population, in addition to the prior eligibility criteria we used, to patients with Medicare A and B, no MC HMO, and age at the time of enrollment ≥ 65 years, enrolled for age not for disability.

We regenerated our data according to the following inclusion criteria: claims whose CLM_TYPE = [40 Outpt, 41 Outpt full encounter, 42 Outpt abbreviated, and 71 RUIC O local carrier non-DMEPOS claim] whose OPSRVTYP = 3 are included for both NCH and Outpatient datasets. A typical NCH file has plcsrvc_nch = [11 Ofc] (Private Practice Office care provider) and plcsrvc_outp = [13 Assisted Living, 22 Outpatient Hospital, 26 Military Treatment Facility, 31 Skilled Nursing Facility, 32 Nursing Facility, 50 Federally Qualified Health Center, 71 State and Local Public Health Clinic, 72 Rural Health Clinic]. A typical Outpatient file has opsrvtype = [3 Institutional Outpatient providers], fac_type_outpt = [1 Hosp, and 2 Skilled Nursing Facility]. This approach was approved by S-M review. 

In the 488 NCH files there were 162,772,263 records. Of these, 93,587,805 records (57.5%) were Private Practice Office records and 18,904,104 records (11.6%) were Institutional Outpatient records. In the 380 Outpatient files, there were 75,218,148 records. Of these, 0 were Private Practice Office records and 57,990,140 records (77.1%) were Institutional Outpatient records. Thus, 75.4% of Inst. OP records originated from the Outpatient files and 24.6% originated from the NCH files, whereas 100% of the PP Office records originated from the NCH files.

The distributions of patients in the Inst. OP dataset were 88.0 W, 7.3 AA and 4.7% other races, and in the PP Office dataset, they were 87.9 W, 7.2 AA and 4.9% other races (Table A1), similar to the Outpatient and NCH dataset values. Most patients had stage I BC in both datasets, with progressively smaller percentages with advancing stages. The distributions of patterns were significantly different between W and AA patients, with AA patients having far fewer early-stage BC cases and higher rates of later-stage BC than W patients in both Inst. OP and PP Office datasets. This trend was analogous to the Outpatient and NCH data, although there were small differences in absolute values.

**Table A1:** Patient distribution by stage.

	Inst. Outpt.							PP Office						
Stage	All	% in Stage	W	% in Stage	AA	% in Stage	AA/ (W+AA × 100%)	All	% in Stage	W	% in Stage	AA	% in Stage	AA/ (W+AA × 100%)
I	125,391	53.1%	112,023	53.9%	7292	42.1%	6.1%	129,105	53.2%	115,191	54.0%	7345	42.3%	6.0%
II	71,862	30.4%	62,544	30.1%	5890	34.0%	8.6%	73,742	30.4%	64,143	30.1%	5924	34.1%	8.5%
III	25,998	11.0%	22,203	10.7%	2675	15.4%	10.8%	26,603	11.0%	22,758	10.7%	2661	15.3%	10.5%
IV	12,947	5.5%	11,021	5.3%	1483	8.6%	11.9%	13,148	5.4%	11,261	5.3%	1431	8.2%	11.3%
Total	236,198		207,791		17,340			242,598		213,353		17,361		

We compared the differences in the distributions of age, comorbidity index, and treatment and AE rates by stage grouping and race between the Inst. OP and the PP Office settings to the differences between the Outpatient and NCH datasets for statistical significance (Table A2). We counted individual treatments multiple times, each time a 0, 1, 2, ..., n AE was recorded. The data confirmed that the age distributions of the Inst. OP and PP Ofc. datasets were higher than in the Outpatient/NCH datasets, corresponding to the inclusion of only patients who qualified by age ≥ 65 years. Overall, the data showed concurrences between the two comparisons in the effects of ER/PR (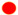), Her2 (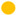) status, and race (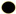) on distribution differences in age, comorbidity index, TR/PT, and AE/TR. The congruence between the Outpatient/NCH and Inst. OP/PP Ofc. datasets (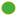) was most evident in files that were annotated for ER/PR status and less so in the files annotated for Her2 that had much fewer numbers.

**Table A2:** Distribution of age, comorbidity index, and treatment and AE rates by stage group race and treatment setting.

	W					AA				
	% ER/PR-	Age	Comb	TR/PT	AE/TR	% ER/PR-	Age	Comb	TR/PT	AE/TR
**Stage I–III**										
**Inst. Outpt.**										
ER/PR+	85.8	75.3 ± 7.2	3.0 ± 3.1	0.6	0.40	75.8 ^a^ 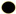	75.0 ± 7.2 ^b^ 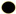	3.5 ± 3.3 ^b^ 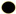	0.9 ^a^ 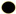	0.39 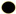
ER/PR-	14.2	75.0 ± 7.3 ^d^ 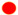	2.7 ± 3.1 ^d^ 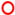	1.5 ^c^ 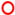	0.43 ^c^ 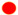	24.4 ^a^ 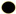	74.0 ± 7.0 ^b,d^ 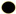 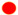	3.1 ± 3.3 ^b,d^ 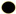 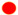	2.2 ^a,c^ 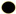 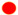	0.43 ^c^ 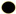 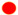
Her2-	89.3	75.0 ± 7.3	3.3 ± 3.0	0.9	0.42	87.4 ^a^ 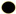	74.6 ± 7.3 ^b^ 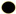	3.8 ± 3.2 ^b^ 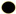	1.3 ^a^ 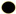	0.43 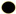
Her2+	10.7	74.7 ± 7.5 ^f^ 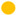	3.4 ± 3.1 ^f^ 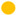	4.7 ^e^ 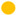	0.40 ^e^ 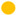	12.6 ^a^ 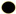	74.3 ± 7.1 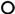 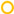	3.6 ± 3.1 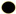 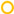	5.5 ^a,e^ 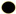 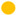	0.36 ^a,e^ 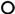 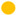
**PP Office**										
ER/PR+	85.8	75.3 ± 7.2 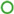	2.7 ± 2.8 ^h^ 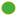	1.8 ^g^ 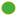	0.52 ^g^ 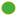	75.7 ^a^ 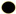	75.0 ± 7.2 ^b^ 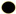 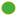	3.1 ± 3.0 ^b,h^ 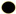 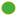	1.6 ^a,g^ 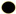 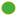	0.58 ^a,g^ 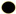 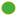
ER/PR-	14.2	75.0 ± 7.2 ^d^ 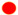 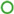	2.4 ± 2.7 ^d,h^ 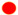 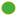	4.2 ^c,g^ 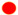 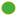	0.60 ^c,g^ 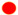 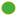	24.3 ^a^ 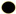	74.0 ± 6.9 ^b,d^ 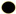 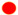 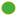	2.8 ± 3.0 ^b,d,h^ 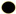 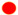 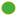	3.4 ^a,c,g^ 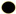 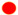 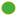	0.59 ^g^ 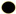 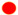 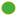
Her2-	89.3	74.9 ± 7.3 ^h^ 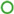	3.1 ± 2.8 ^h^ 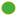	1.6 ^g^ 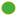	0.48 ^g^ 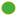	87.5 ^a^ 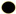	74.6 ± 7.2 ^b^ 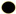 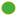	3.6 ± 3.0 ^b,h^ 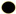 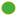	1.4 ^a^ 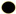 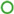	0.52 ^a,g^ 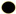 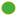
Her2+	10.7	74.5 ± 7.5 ^f^ 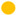 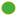	3.1 ± 2.8 ^h^ 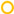 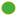	7.3 ^e,g^ 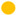 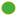	0.49 ^e,g^ 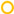 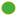	12.5 ^a^ 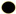	74.3 ± 7.1 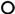 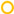 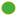	3.4 ± 2.9 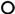 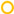 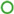	4.5 ^a,e,g^ 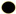 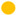 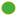	0.46 ^a,e,g^ 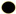 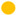 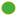
**Stage IV**										
**Inst. Outpt.**										
ER/PR+	79.4	76.1 ± 7.5	2.0 ± 2.8	3.0	0.37	68.0 ^a^ 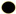	75.1 ± 7.4 ^b^ 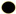	2.1 ± 2.9 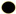	2.9 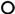	0.39 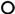
ER/PR-	20.6	75.8 ± 7.5 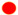	1.7 ± 2.8 ^d^ 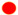	3.0 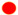	0.37 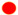	32.0 ^a^ 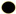	74.6 ± 6.8 ^b^ 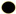 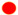	1.8 ± 2.9 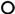 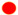	2.8 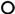 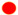	0.42 ^a^ 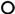 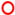
Her2-	80.6	75.9 ± 7.8	3.1 ± 3.0	4.2	0.40	79.0 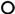	75.0 ± 7.6 ^b^ 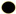	3.2 ± 3.1 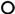	3.9 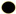	0.42 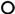
Her2+	19.4	75.3 ± 7.7 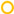	3.1 ± 3.1 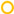	8.4 ^e^ 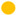	0.32 ^e^ 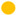	21.0 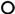	74.6 ± 7.6 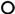 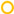	3.1 ± 3.2 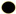 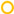	4.0 ^a^ 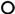 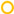	0.25 ^a,e^ 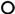 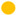
**PP Office**										
ER/PR+	79.2	76.2 ± 7.5 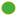	1.8 ± 2.6 ^h^ 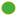	6.4 ^g^ 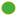	0.57 ^g^ 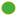	68.1 ^a^ 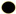	75.2 ± 7.4 ^b^ 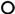 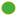	2.0 ± 2.7 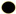 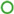	4.7 ^a,g^ 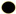 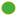	0.59 ^g^ 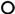 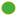
ER/PR-	20.8	75.9 ± 7.5 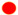 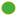	1.6 ± 2.6 ^d^ 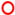 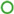	7.7 ^c,g^ 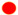 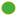	0.6 ^c,g^ 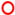 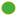	31.9 ^a^ 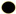	75.2 ± 6.8 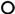 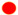 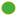	1.6 ± 2.7 ^d^ 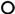 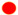 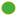	4.3 ^a,g^ 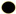 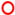 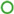	0.64 ^g^ 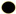 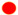 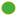
Her2-	80.4	76.0 ± 7.8 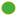	2.8 ± 2.8 ^h^ 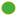	3.9 ^g^ 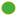	0.51 ^g^ 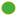	78.7 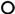	75.2 ± 7.5 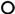 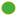	3.1 ± 2.9 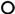 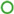	3.9 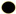 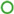	0.55 ^g^ 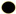 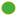
Her2+	19.6	75.3 ± 7.7 ^f^ 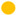 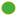	2.8 ± 2.9 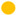 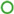	12.8 ^e,g^ 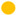 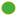	0.54 ^e,g^ 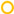 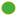	21.3 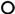	74.8 ± 7.8 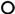 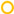 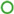	2.8 ± 2.8 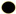 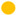 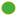	3.3 ^a^ 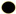 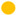 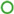	0.38 ^a,e,g^ 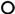 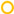 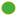

**Legend.** Statistically significant difference between: AA and W patients by ^a^ Chi-square or ^b^ *t*-test; ER/PR+ and ER/PR- by ^c^ Chi-square or ^d^ *t*-test; Her2- and Her2+ by ^e^ Chi-square or ^f^ *t*-test; Inst. Outpatient and PP Office by ^g^ Chi-square or ^h^ *t*-test. Correlations between Outpatient file and Institutional Outpatient data and between NCH file and Private Practice Office data. Correlation of the race effect (Yes 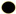/No 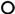), ER/PR effect (Yes 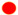/No 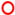), Her2 effect (Yes 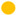/No 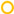), and venue effect (Yes 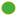/No 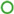).

Overall, the data showed congruity between the general conclusions derived from comparing the Outpatient to the NCH and Inst. OP to the PP Ofc. datasets, with some exceptions where the number of entries was relatively small. The most striking conclusions, however, were the agreements between the Outpatient and NCH patterns compared with the Inst. OP and the PP Ofc. patterns in the four values of age, comorbidity index, TR/PT, and AE/TR in both W and AA patients designated by the green solid dots. Some of the stage IV categories that had more variability and fewer entries were less congruent than the stage I–III categories. We did not compare the adverse event categories in the two datasets for statistical differences. They remained similar in distribution, which varied slightly according to race, as in the primary study.

We also included additional acknowledgements used to generate the S-M datasets.

The authors apologize for any inconvenience caused. The authors state that the scientific conclusions are unaffected. This correction was approved by the Academic Editor. The original publication has also been updated.

**Acknowledgments:** This study used the linked SEER-Medicare database. The interpretation and reporting of these data are the sole responsibility of the authors. The authors acknowledge the efforts of the National Cancer Institute; Information Management Services (IMS), Inc.; and the Surveillance, Epidemiology, and End Results (SEER) Program tumor registries in the creation of the SEER-Medicare database and wish to thank them for their advice and review of the datasets designating the different treatment venues. The collection of cancer incidence data from the California Cancer Registry used in this study was supported by the California Department of Public Health pursuant to California Health and Safety Code Section 103885; Centers for Disease Control and Prevention’s (CDC) National Program of Cancer Registries, under cooperative agreement 1NU58DP007156; the National Cancer Institute’s Surveillance, Epidemiology and End Results Program under contract HHSN261201800032I awarded to the University of California, San Francisco, contract HHSN261201800015I awarded to the University of Southern California, and contract HHSN261201800009I awarded to the Public Health Institute. The ideas and opinions expressed herein are those of the author(s) and do not necessarily reflect the opinions of the State of California, Department of Public Health, the National Cancer Institute, and the Centers for Disease Control and Prevention or their Contractors and Subcontractors.
