# Supplementary material for: Racial Disparities in Breast Cancer Treatments and Adverse Events in the SEER-Medicare Data
Source: Cancers (Basel). 2023 Aug 30;15(17):4333. doi: 10.3390/cancers15174333 (PMC10486612; doi:10.3390/cancers15174333)
Supplement: Supplementary file 1 [file cancers-15-04333-s001.zip › cancers-2551300-supplementary.pdf]

Supplementary Tables

Table S1. Distribution of patient characteristics, tumor markers, treatments and AEs in stage I-III Outpatient dataset patients.

Stage I-III										
Outpatient										
	Patients	%	Age	SD	COMB	SD	TR	TR/PT	AE	AE/TR
All	243,814		67.6	11.0	4.2	2.8	224,923	0.9	84,390	0.38
ER/PR+	185,236	83.1%	68.3	10.6	4.2	2.8	149,715	0.8	55,023	0.37
ER/PR-	37,572	16.9%	65.0	11.6	4.0	4.0	64,158	1.7	25,544	0.40
ER/PR +/- Total	222,808	100.0%								
%ER/PR annotated	91.4%									
Her2-	62,266	87.8%	71.3	9.5	4.1	2.8	61,957	1.0	24,728	0.40
Her2+	8,682	12.2%	69.4	10.6	4.0	2.9	38,424	4.4	14,487	0.38
Her2-/+ Total	70,948	100.0%								
% Her2 annotated	29.1%									
W										
All	205,328		68.0	10.8	4.2	2.8	172,130	0.8	64,412	0.37
ER/PR+	158,847	84.6%	68.6	10.5	4.2	2.8	119,177	0.8	43,745	0.37
ER/PR-	28,870	15.4%	65.8	11.4	4.0	2.8	45,101	1.6	17,893	0.40
ER/PR +/- Total	187,717	100.0%								
%ER/PR annotated	91.4%									
Her2-	51,500	88.3%	71.8	9.2	4.1	2.8	47,397	0.9	18,868	0.40
Her2+	6,838	11.7%	70.2	10.2	4.0	2.9	29,439	4.3	11,283	0.38
Her2-/+ Total	58,338	100.0%								
% Her2 annotated	28.4%									
AA										
All	24,664		64.6	12.3	4.5	2.9	40,931	1.7	15,952	0.39
ER/PR+	15,933	71.1%	65.9	12.1	4.5	2.9	22,341	1.4	8,558	0.38
ER/PR-	6,473	28.9%	61.8	12.3	4.2	2.9	15,858	2.4	6,425	0.41
ER/PR +/- Total	22,406	100.0%								
%ER/PR annotated	90.8%									
Her2-	7,117	85.3%	68.0	11.4	4.4	3.0	12,037	1.7	4,932	0.41
Her2+	1,226	14.7%	65.4	12.1	4.1	3.0	6,750	5.5	2,551	0.38
Her2-/+ Total	8,343	100.0%								
% Her2 annotated	33.8%									
Legend: AA: African American patients, AE: adverse events, COMB: comorbidites, ER: estrogen receptor,										
Her2: Human Epidermal Growth Facaor Receptor 2, PT: patients, SD: standard deviation, TR: treatment, W: white patients										

Table S2. Distribution of patient characteristics, tumor markers, treatments and AEs in stage I-III NCH dataset patients.

Stage I-III										
NCH										
	Patients	%	Age	SD	COMB	SD	TR	TR/PT	AE	AE/TR
All	279,814		67.4	11.0	3.7	2.7	615,865	2.2	331,746	0.54
ER/PR+	212,751	83.2%	68.1	10.6	3.6	2.7	408,470	1.9	213,869	0.52
ER/PR-	43,099	16.8%	64.9	11.6	3.5	2.7	158,099	3.7	89,816	0.57
ER/PR +/- Total	255,850	100.0%								
%ER/PR annotated	91.4%									
Her2-	71,962	87.9%	71.2	9.4	3.6	2.8	108,378	1.5	54,147	0.50
Her2+	9,925	12.1%	69.5	10.4	3.5	2.8	61,036	6.1	30,279	0.50
Her2-/+ Total	81,887	100.0%								
% Her2 annotated	29.3%									
W										
All	234,768		67.8	10.8	3.6	2.7	523,677	2.2	279,242	0.53
ER/PR+	181,725	84.6%	68.4	10.5	3.6	2.7	356,643	2.0	184,905	0.52
ER/PR-	33,014	15.4%	65.6	11.4	3.5	2.6	126,816	3.8	71,920	0.57
ER/PR +/- Total	214,739	100.0%								
%ER/PR annotated	91.5%									
Her2-	59287	88.4%	71.7	9.1	3.6	2.7	91,285	1.5	45,204	0.50
Her2+	7,812	11.6%	70.2	10.0	3.5	2.8	51,289	6.6	25,388	0.49
Her2-/+ Total	67,099	100.0%								
% Her2 annotated	28.6%									
AA										
All	27,743		64.6	12.2	4.0	2.9	56,982	2.1	32,322	0.57
ER/PR+	17,964	71.2%	65.9	11.9	4.1	2.9	30,486	1.7	17,210	0.56
ER/PR-	7,258	28.8%	61.9	12.2	3.9	2.8	20,709	2.9	11,733	0.57
ER/PR +/- Total	25,222	100.0%								
%ER/PR annotated	90.9%									
Her2-	8,125	85.5%	68.0	11.2	4.0	3.0	11,146	1.4	5,943	0.53
Her2+	1,374	14.5%	65.7	11.9	3.8	2.9	5,090	3.7	2,315	0.45
Her2-/+ Total	9,499	100.0%								
% Her2 annotated	34.2%									
Legend: AA: African American patients, AE: adverse events, COMB: comorbidites, ER: estrogen receptor,										
Her2: Human Epidermal Growth Facaor Receptor 2, PT: patients, SD: standard deviation, TR: treatment, W: white patients										

Table S3. Distribution of patient characteristics, tumor markers, treatments and AEs in stage IV Outpatient dataset patients.

Stage IV										
Outpatient										
	Patients	%	Age	SD	COMB	SD	TR	TR/PT	AE	AE/TR
All	8,664		68.6	12.4	3.8	2.9	44,102	5.1	15,203	0.34
ER/PR+	6,251	80.3%	68.5	12.4	3.8	2.8	32,545	5.2	11,256	0.35
ER/PR-	1,534	19.7%	68.0	12.5	3.9	3.0	9,058	5.9	3,108	0.34
ER/PR +/- Total	7,785	100.0%								
%ER/PR annotated	89.9%									
Her2-	3,039	76.7%	70.7	11.4	4.0	2.9	13,445	4.4	5,099	0.38
Her2+	923	23.3%	67.3	12.9	3.6	2.9	7,863	8.5	2,228	0.28
Her2-/+ Total	3,962	100.0%								
% Her2 annotated	45.7%									
W										
All	6,947		69.4	12.1	3.8	2.8	34,454	5.0	11,834	0.34
ER/PR+	5,109	81.6%	69.3	12.0	3.8	2.8	25,996	5.1	8,902	0.34
ER/PR-	1,149	18.4%	68.6	12.3	3.8	3.0	6,295	5.5	2,196	0.35
ER/PR +/- Total	6,258	100.0%								
%ER/PR annotated	90.1%									
Her2-	2,435	77.3%	71.4	11.0	4.0	2.8	10,786	4.4	4,062	0.38
Her2+	714	22.7%	68.3	12.4	3.6	2.9	6,084	8.5	1,715	0.28
Her2-/+ Total	3,149	100.0%								
% Her2 annotated	45.3%									
AA										
All	1,293		65.3	13.4	4.0	2.9	7,483	5.8	2,817	0.38
ER/PR+	824	72.3%	65.1	13.6	3.9	2.9	5,012	6.1	1,990	0.40
ER/PR-	316	27.7%	65.2	13.1	4.2	3.1	2,165	6.9	727	0.34
ER/PR +/- Total	1,140	100.0%								
%ER/PR annotated	88.2%									
Her2-	455	73.7%	68.0	12.2	4.3	3.1	2,069	4.5	865	0.42
Her2+	162	26.3%	63.4	14.0	3.5	2.7	1,358	8.4	390	0.29
Her2-/+ Total	617	100.0%								
% Her2 annotated	47.7%									

Legend: AA: African American patients, AE: adverse events, COMB: comorbidites, ER: estrogen receptor, Her2: Human Epidermal Growth Facaor Receptor 2, PT: patients, SD: standard deviation, TR: treatment, W: white patients

Table S4. Distribution of patient characteristics, tumor markers, treatments and AEs in stage IV NCH dataset patients.

Stage IV										
NCH										
	Patients	%	Age	SD	COMB	SD	TR	TR/PT	AE	AE/TR
All	9,913		68.9	12.3	3.5	2.7	91,106	9.2	50,181	0.55
ER/PR+	7,133	80.3%	68.8	12.3	3.4	2.7	64,368	9.0	35,506	0.55
ER/PR-	1,755	19.7%	68.4	12.3	3.6	2.9	18,320	10.4	10,175	0.56
ER/PR +/- Total	8,888	100.0%								
%ER/PR annotated	89.7%									
Her2-	3,553	77.2%	70.9	11.2	3.6	2.8	14,480	4.1	7,584	0.52
Her2+	1,047	22.8%	67.7	12.7	3.3	2.8	11,812	11.3	6,210	0.53
Her2-/+ Total	4,600	100.0%								
% Her2 annotated	46.4%									
W										
All	7,942		69.5	12.0	3.5	2.7	78,805	9.9	43,512	0.55
ER/PR+	5,817	81.6%	69.5	11.9	3.4	2.7	56,953	9.8	31,688	0.56
ER/PR-	1,310	18.4%	69.0	12.1	3.5	2.8	14,306	10.9	7,851	0.55
ER/PR +/- Total	7,127	100.0%								
%ER/PR annotated	89.7%									
Her2-	2,843	77.9%	71.6	10.9	3.5	2.8	11,603	4.1	6,096	0.53
Her2+	808	22.1%	68.7	12.2	3.3	2.8	10,577	13.1	5,534	0.52
Her2-/+ Total	3,651	100.0%								
% Her2 annotated	46.0%									
AA										
All	1,468		65.6	13.1	3.7	2.8	7,927	5.4	4,214	0.53
ER/PR+	946	72.7%	65.4	13.3	3.5	2.8	5,023	5.3	2,629	0.52
ER/PR-	356	27.3%	65.6	12.8	3.9	2.9	2,289	6.4	1,233	0.54
ER/PR +/- Total	1,302	100.0%								
%ER/PR annotated	88.7%									
Her2-	531	74.4%	68.1	11.9	3.8	3.0	2,042	3.8	1,087	0.53
Her2+	183	25.6%	64.0	13.8	3.4	2.7	602	3.3	293	0.49
Her2-/+ Total	714	100.0%								
% Her2 annotated	48.6%									
Legend: AA: African American patients, AE: adverse events, COMB: comorbidites, ER: estrogen receptor,										
Her2: Human Epidermal Growth Facaor Receptor 2, PT: patients, SD: standard deviation, TR: treatment, W: white patients										

Table S5. Treatments associated with common AE differences in Stage I-III Outpatient vs. NCH.

**Outpatient**					**NCH**				
**Nausea/vomiting -** 26.1% of AEs					**Nausea/vomiting -** 53.4% of AEs				
Treatment	Pts.	Age + SD	Comorbidity Index + SD	TR Freq	Treatment	Pts.	Age + SD	Comorbidity Index + SD	TR Freq
Taxane	5,429	62.9 + 10.4	4.0 + 2.5	24.6%	Taxanes	42,133	65.5 + 9.6	4.0 + 2.4	23.8%
Anthracyclines	3,415	63.1 + 9.4	3.9 + 2.3	15.5%	Anthracyclines	31,239	66.0 + 8.5	4.2 + 2.3	17.7%
Her2 targeting agts	3,187	62.4 + 11.6	4.0 + 2.5	14.4%	Pyrimidine analogs	24,838	66.0 + 10.5	4.5 + 2.5	14.0%
Platinum cmpds.	2,613	61.9 + 11.2	3.7 + 2.3	11.8%	Her2 targeting agts.	20,700	65.9 + 10.3	4.2 + 2.5	11.7%
Pyrimidine analogs	1,907	60.3 + 12.4	3.9 + 2.5	8.6%	Alkylating agents	15,713	66.8 + 8.4	4.1 + 2.4	8.9%
Alkylating agents	1,897	64.7 + 9.4	4.1 + 2.5	8.6%	Platinum cmpds	14,367	64.7 + 10.2	4.1 + 2.4	8.1%
Total tallied	18,448			83.6%	Folate analogs	9,248	69.1 + 8.7	5.0 + 2.5	5.2%
					Total tallied	158,238			89.4%
Mean+SD TR pts		62.3 + 11.2	4.1 + 2.5				65.9 + 9.7	4.3 + 2.4	

**Outpatient**					**NCH**				
**Weakness/malaise, delirium -** 23.4% of AEs					**Weakness/malaise, delirium** – 8.9% of AEs				
Treatment	Pts	Age + SD	Comorbidity Index + SD	TR Freq	Treatment	Pts.	Age + SD	Comorbidity Index + SD	TR Freq
Taxanes	4,233	63.7 + 10.4	4.0 +2.5	21.4%	Her2 targeting agts.	6,783	66.5 + 10.7	4.5 + 2.5	22.9%
Her2 targeting agts.	4,082	64.7 + 11.3	4.2 + 2.6	20.6%	Taxanes	5,261	65.2 + 9.6	4.4 + 2.5	17.8%
Bisphosphonates	2,739	62.9 + 11.8	4.6 + 2.6	13.8%	Bisphosphonates	4,838	66.3 + 11.1	4.8 + 2.5	16.3%
Anthracyclines	1,947	64.4 + 9.0	4.0 + 2.3	9.8%	Antiestrogens	2,528	66.4 + 10.5	4.9 + 2.4	8.5%
Platinum cmpds.	1,741	62.7 + 11.6	4.1 + 2.3	8.8%	Anthracyclines	2,230	65.0. + 8.8	4.6 + 2.3	7.5%
Antiestrogen	1,313	63.6 + 13.2	5.1 + 2.8	6.6%	Pyrimidine analogs	2,115	65.7 + 10.2	4.7 + 2.5	7.1%
Total tallied	16,055			81.2%	Platinum cmpds.	1,672	65.1 + 10.4	4.4 + 2.5	5.6%
					Total tallied	25,427			85.9%
Mean+SD TR pts		64.1 + 11.2	4.4 + 2.6				66.9 + 10.5	4.8 + 2.5	

**Outpatient**					**NCH**				
**Neutropenia/Leukopenia -** 15.7% of AEs					**Neutropenia/Leukopenia** - 8.8% of AEs				
Treatment	Patients	Age + SD	Comorbidity Index + SD	TR Freq	Treatment	Pts.	Age + SD	Age + SD	TR Freq
Taxanes	4,225	63.9 + 10.5	3.9 + 2.4	31.8%	Taxanes	8,132	66.0 + 9.2	4.0 + 2.4	27.7%
Alkylating agents	2,203	65.6 + 9.4	4.1 + 2.4	16.6%	Anthracyclines	4,599	65.8 + 8.6	4.0 + 2.3	15.7%
Her2 targeting agts.	1,796	64.2 + 10.6	4.1 + 2.4	13.5%	Her2 targeting agts.	4,507	65.9 + 10.1	4.1 + 2.4	15.4%
Anthracyclines	1,607	63.8 + 9.6	4.0 + 2.3	12.1%	Platinum cmpds.	2,881	65.3 + 9.9	4.1 + 2.4	9.8%
Platinum cmpds.	1,035	63.5 + 10.7	4.0 + 2.4	7.8%	Alkylating agents	2,847	66.5 + 8.6	3.9 + 2.4	9.7%
Pyrimidine analogs	880	62.1 + 11.9	4.1 + 2.5	6.6%	Pyrimidine analogs	2,245	64.4 + 12.0	4.3 + 2.5	7.7%
Total tallied	11,746			88.5%	Total tallied	25,211			86.0%
Mean+SD TR pts		63.7 + 10.6	4.0 + 2.4				65.6 + 9.6	4.0 + 2.4	

**Outpatient**					**NCH**				
**Electrolyte abnormalities** – 4.5% of AEs					**Electrolyte abnormalities** – 9.3% of AEs				
Treatment	Pts.	Age + SD	Comorbidity Index + SD	TR Freq	Treatment	Pts	Age + SD	Comorbidity Index + SD	TR Freq
Taxanes	1,167	64.1 + 10.7	4.1 + 2.5	30.6%	Taxanes	7,116	64.7 + 10.1	4.2 + 2.4	23.0%
Her2 targeting agts.	657	64.5 + 10.9	4.2 + 2.4	17.2%	Her2 targeting agts.	5,701	65.3 + 10.9	4.3 + 2.5	18.5%
Alkylating agents	472	66.5 + 9.2	4.0 + 2.5	12.4%	Pyrimidine analogs	3,345	63.3 + 11.8	4.4 + 2.6	10.8%
Platinum cmpds.	355	63.6 + 11.4	4.0 + 2.3	9.3%	Alkylating agenta	2,521	65.9 + 9.3	4.2 + 2.5	8.2%
Anthracyclines	327	63.8 + 9.5	3.8 + 2.3	8.6%	Anthracyclines	2,492	65.0 + 9.3	4.1 + 2.4	8.1%
Bisphosphonates	252	61.6 + 12.5	4.1 + 2.5	6.6%	Platinum cmpds.	2,205	64.0 +10.7	4.2 + 2.3	7.1%
Pyrimidine analogs	214	58.6 + 13.9	3.9 + 2.4	5.6%	Vinca alkaloids	1,974	59.9 + 12.5	3.7 + 2.5	6.4%
					Bisphosphonates	1,765	62.4 + 11.7	4.4 + 2.7	5.7%
Total tallied	3,444			90.3%	Total tallied	27,119			87.8%
Mean+SD TR pts		63.5 + 11.6	4.3 + 2.6				65.1 + 10.5	4.3 + 2.5	

Table S6. Treatments associated with common AE differences in Stage IV Outpatient v. NCH sets.

**Outpatient**					**NCH**				
**Weakness/ malaise, delirium** - 24.5% of AEs					**Weakness/malaise, delirium** – 11.9% of AEs				
Treatment	Pis	Age + SD	Comorbidity Index + SD	TR Freq	Treatment	Pts	Age + SD	Comorbidity Index + SD	TR Freq
Bisphosphonates	1,098	64.8 + 12.6	4.1 + 2.9	29.4%	Bisphosphonates	1,579	67.4 + 11.0	4.4 + 2.8	26.3%
Her2 targeting agts.	719	63.3 + 11.0	3.3 + 2,5	19.3%	Her2 targeting agts.	1,196	65.5 + 12.6	4.1 + 2.6	20.0%
Taxanes	661	63.6 + 11.7	3.6 + 2.5	17.7%	Taxane	929	66.6 + 10.7	4.5 + 2.8	15.5%
Antiestrogens	363	69.5 + 12.2	4.8 + 2.9	9.7%	Antiestrogen	582	68.9 + 10.5	4.9 + 2.7	9.7%
Platinum cmpds.	235	60.3. + 12.3	3.5 + 2.6	6.3%	Pyrimidine analogs	542	65.1 + 11.0	4.1 + 2.5	9.0%
Pyrimidine analogs	165	63.5 + 13.1	3.7 + 2.8	4.4%	Platinum cmpds.	248	65.3 + 11.3	3.7 + 1.9	4.1%
	3,241			86.9%	Total tallied	5,076			84.7%
Mean+SD TR pts		64.8 + 11.9	3.9 + 2.8		Mean+SD TR pts		67.0 + 11.3	4.3 + 2.7	

**Outpatient**					**NCH**				
**Nausea/vomiting** - 22.0% of AEs					**Nausea/vomiting** – 45.7% of AEs				
Treatment	Pts.	Age + SD	Comorbidity Index + SD	TR Freq	Treatment	Pts	Age + SD	Comorbidity Index + SD	TR Freq
Taxanes	715	63.3 +11.6	3.7 + 2.6	21.4%	Taxanes	6,221	65.2 + 10.8	3.6 + 2.5	27.1%
Bisphosphonates	668	61.6 + 13.3	3.7 + 2.5	20.0%	Her2 targeting agts.	4,071	64.3 + 11.1	3.5 + 2.5	17.7%
Her2 targeting agts.	563	61.1 + 12.5	4.0 + 2.7	16.8%	Pyrimidine analogs	2,659	63.8 + 11.1	3.4 + 2.4	11.6%
Platinum cmpds.	345	61.5 + 11.3	3.4 + 2.7	10.3%	Bisphosphonates	2,345	64.3 + 11.6	3.7 + 2.6	10.2%
Pyrimidine analogs	338	61.9 + 10.6	3.7 + 2.8	10.1%	Platinum cmpds	1,775	64.9 + 10.2	3.6 + 2.5	7.7%
Anthracyclines	172	60.6 + 11.2	3.5 + 2.7	5.1%	Vinca alkaloids	1,376	63.9 + 12.0	3.4 + 2.4	6.0%
Antiestrogens		65.5 + 10.9	4.0 + 2.5	4.8%	Anthracyclines	1,233	65.2 + 10.2	3.6 + 2.1	5.4%
Total tallied	2,960			88.6%	Total tallied	18,680			85.8%
Mean+SD TR pts		62.4 + 12.2	3.8 + 2.7		Mean+SD TR pts		65.1 + 11.3	3.6 + 2.5	

**Outpatient**					**NCH**				
**Neutropenia/Leukopenia** - 12.0% of AEs					**Neutropenia/Leukopenia** – 7.2% of AEs				
Treatment	Pts.	Age + SD	Comorbidity Index + SD	TR Freq	Treatment	Pts.	Age + SD	Comorbidity Index + SD	TR_Fr eq
Taxanes	561	61.5 + 11.9	3.3 + 2.5	30.7%	Taxane	965	64.8 + 11.4	3.7 + 2.6	26.9%
Pyrimidine an.	222	60.7 + 11.8	3.2 + 2.6	12.1%	Her2 targeting agts	653	63.7 + 11.2	3.5 + 2.3	18.2%
Bisphosphonates	217	62.2 + 11.2	3.4 + 2.3	11.9%	Bisphosphonates	382	64.0 + 11.8	3.3 + 2.3	10.6%
Her2 targeting agts.	179	58.8 + 13.1	3.2 + 2.2	9.8%	Pyrimidine analogs	326	63.3 + 10.9	3.5 + 2.6	9.1%
Platinum cmpds.	132	60.6 + 13.3	3.1 + 2.6	7.2%	Vinca alkaloids	321	63.9 + 11.7	3.7 + 2.4	8.9%
Anthracyclines	110	60.7 + 11.9	3.0 + 2.4	6.0%	Platinum cmpd	291	64.6 + 10.8	3.5 + 2.5	8.1%
Alkylating agents	108	60.9 + 11.3	3.3 + 2.3	5.9%	Anthracyclines	201	65.7 + 9.2	3.5 + 2.4	5.6%
Vinca alkaloids	103	59.4 + 12.7	3.1 + 2.6	5.6%	VEGF inhibitor	198	62.3 + 10.7	3.3 + 2.4	5.5%
Total tallied	1,632			89.3%	Total tallied	3,337			93.0%
Mean+SD TR pts		61.5 + 11.9	3.2 + 2.5		Mean+SD TR pts		65.0 + 11.0	3.6 + 2.5	

**Outpatient**					**NCH**				
**Electrolyte abnormalities** – 4.1% of AEs					**Electrolyte abnormalities** – 12.9% of AEs				
Treatment	Pts.	Age + SD	Comorbidity Index + SD	TR_Fr eq	Treatment	Pts.	Age + SD	Comorbidity Index + SD	TR Freq
Taxanes	1,167	64.1 + 10.7	4.1 + 2.5	30.6%	Her2 targeting agts.	1,406	64.4 + 12.0	4.0 + 2.5	21.7%
Her2 targeting agts.	657	64.5 + 10.9	4.2 + 2.4	17.2%	Taxanes	1,141	64.4 + 10.9	4.0 + 2.7	17.6%
Alkylating agents	472	66.5 + 9.2	4.0 + 2.5	12.4%	Pyrimidine analogs	873	63.5 + 12.3	3.6 + 2.3	13.4%
Platinum cmpds.	355	63.6 +11.4	4.0 + 2.3	9.3%	Bisphosphonates	706	65.0 + 12.0	4.0 + 2.6	10.9%
Anthracyclines	327	63.8 + 9.5	3.8 + 2.3	8.6%	Vinca alkaloids	393	63.8 + 11.8	4.0 + 2.4	6.1%
Bisphosphonates	252	61.6 +12.5	4.1 +2.5	6.6%	VEGF inhibitors	394	64.9 + 11.2	3.8 + 2.6	6.1%
Pyrimidine analogs	214	58.6 + 13.9	3.9 + 2.4	5.6%	Folate analogs	340	62.8 + 11.8	3.3 + 1.5	5.2%
					Antiestrogens	331	68.4 + 9.6	4.3 + 2.6	5.1%
Total tallied	3,444			90.3%		5,584			86.0%
Mean+SD TR pts		64.9 + 11.6	4.1 + 2.8		Mean+SD TR pts		65.2 + 11.6	3.9 + 2.6	

Table S7. Race distribution of the most frequent AEs in stage I-III Outpatient and NCH patients.

**Stage I-III**					
**Outpatient**					
**White Patients**			**AA patients**		
AEs	AEs with TRs	% of all TR AEs	AEs	AEs with TRs	% of all TR AEs
Nausea/vomiting	16,689	25.9%	Nausea/vomiting	4,462	28.0%
Weakness/ malaise, delirium	15,803	24.5%	Weakness/ malaise, delirium	3,100	19.4%
Neutropenia/Leukopenia	9,805	15.2%	Neutropenia/Leukopenia	2,747	17.2%
Respiratory symptoms	4,948	7.7%	Respiratory symptoms	1,208	7.6%
Infection/fever	3,173	4.9%	Anemias	1,146	7.2%
Anemias	3,058	4.7%	Infection/fever	737	4.6%
Electrolyte abnormalities	2,872	4.5%	Electrolyte abnormalities	721	4.5%
Diarrhea	2,303	3.6%	Thrombophilia	541	3.4%
Thrombophilia	1,945	3.0%	Diarrhea	453	2.8%
Constipation	1,715	2.7%	Constipation	407	2.6%
Pulmonary Embolus	936	1.5%	Weight loss/ malnutrition	191	1.2%
Weight loss/ malnutrition	637	1.0%	Pulmonary Embolus	180	1.1%
Total tallied	63,884	99.2%	Total tallied	15,893	99.6%
**NCH**					
**White Patients**			**AA patients**		
AEs	AEs with TRs	% of all TR AEs	AEs	AEs with TRs	% of all TR AEs
Nausea/vomiting	150,199	53.8%	Nausea/vomiting	16,677	51.6%
Weakness/ malaise, delirium	25,975	9.3%	Anemias	3,718	11.5%
Neutropenia/Leukopenia	24,810	8.9%	Electrolyte abnormalities	3,627	11.2%
Electrolyte abnormalities	23,501	8.4%	Neutropenia/Leukopenia	3,187	9.9%
Anemias	21,080	7.5%	Weakness/ malaise, delirium	2,132	6.6%
Respiratory symptoms	10,178	3.6%	Respiratory symptoms	1,035	3.2%
Infection/fever	8,781	3.1%	Infection/fever	816	2.5%
Thrombophilia	4,939	1.8%	Thrombophilia	442	1.4%
Diarrhea	2,904	1.0%			
Total tallied	272,367	97.5%	Total tallied	31,634	97.9%

Table S8. Race distribution of the most frequent AEs in stage IV Outpatient and NCH patients.

Stage IV					
**Outpatient**					
**White Patients**			**AA patients**		
AEs	AEs with TRs	% of all TR AEs	AEs	AEs with TRs	% of all TR AEs
Weakness/ malaise, delirium	2,919	24.7%	Weakness/ malaise, delirium	647	23.0%
Nausea/vomiting	2,699	22.8%	Nausea/vomiting	584	20.7%
Neutropenia/Leukopenia	1,323	11.2%	Neutropenia/Leukopenia	415	14.7%
Respiratory symptoms	1,101	9.3%	Respiratory symptoms	247	8.8%
Anemias	806	6.8%	Anemias	243	8.6%
Thrombophilia	701	5.9%	Infection/fever	164	5.8%
Infection/fever	650	5.5%	Electrolyte abnormalities	134	4.8%
Electrolyte abn.	479	4.0%	Diarrhea	94	3.3%
Constipation	337	2.8%	Constipation	94	3.3%
Pulmonary Embolus	330	2.8%	Thrombophilia	83	2.9%
Diarrhea	311	2.6%	Pulmonary Embolus	70	2.5%
			Weight loss/ malnutrition	36	1.3%
Total tallied	11,656	98.5%	Total tallied	2,811	99.8%
**NCH**					
**White Patients**			**AA patients**		
AEs	AEs with TRs	% of all TR AEs	AEs	AEs with TRs	% of all TR AEs
Nausea/vomiting	20317	46.7%	Nausea/vomiting	1714	40.7%
Weakness/ malaise, delirium	5285	12.1%	Electrolyte abnormalities	715	17.0%
Electrolyte abnormalities	5162	11.9%	Anemias	507	12.0%
Anemias	3880	8.9%	Neutropenia/Leukopenia	409	9.7%
Neutropenia/Leukopenia	2996	6.9%	Weakness/ malaise, delirium	401	9.5%
Respiratory symptoms	2196	5.0%	Respiratory symptoms	196	4.7%
Infection/fever	1001	2.3%	Infection/fever	97	2.3%
Thrombophilia	984	2.3%	Pulmonary Embolus	69	1.6%
Pulmonary Embolus	552	1.3%			
Diarrhea	499	1.1%			
Total tallied	42872	98.5%	Total tallied	4108	97.5%
